# Difference in microRNA expression and editing profile of lung tissues from different pig breeds related to immune responses to HP-PRRSV

**DOI:** 10.1038/srep09549

**Published:** 2015-04-09

**Authors:** Jia Li, Zhisheng Chen, Junlong Zhao, Liurong Fang, Rui Fang, Jiang Xiao, Xing Chen, Ao Zhou, Yingyin Zhang, Liming Ren, Xiaoxiang Hu, Yaofeng Zhao, Shujun Zhang, Ning Li

**Affiliations:** 1 State Key Laboratory for Agrobiotechnology, China Agricultural University, Beijing, 100193, People's Republic of China; 2 Key Laboratory of Agricultural Animal Genetics, Breeding and Reproduction of Ministry of Education, Huazhong Agricultural University, Wuhan, 430070, People's Republic of China

## Abstract

Porcine reproductive and respiratory syndrome (PRRS) is one of the most devastating diseases for the pig industry. Our goal was to identify microRNAs involved in the host immune response to PRRS. We generated microRNA expression profiles of lung tissues from Tongcheng or Landrace pigs infected with a highly pathogenic PRRS virus (PRRSV) at 3, 5, 7 dpi (day post infection) and control individuals from these two breeds. Our data showed that 278 known and 294 novel microRNAs were expressed in these combined microRNA transcriptomes. Compared with control individuals, almost half of the known microRNAs (116 in Tongcheng and 153 in Landrace) showed significantly differential expression (DEmiRNAs) at least once. The numbers of down-regulated DEmiRNAs were larger than the corresponding number of up-regulated DEmiRNAs in both breeds. Interestingly, miR-2320-5p, which was predicted to bind to conserved sequences of the PRRSV genome, was down-regulated significantly at 3 dpi after PRRSV infection in both breeds. In addition, PRRSV infection induced a significant increase of microRNA editing level in both breeds. Our results provide novel insight into the role of microRNA in response to PRRSV infection *in vivo*, which will aid the research for developing novel therapies against PRRSV.

Porcine reproductive and respiratory syndrome (PRRS) causes considerable loss in the pig industry worldwide. PRRS virus (PRRSV) is a positive, single-stranded RNA virus, belonging to *Arteriviridae* family, *Nidovirales* order. Infection of PRRSV in pigs causes reproductive failure in sows, respiratory disease in piglets and grower pigs, anorexia, lethargy etc^1^,^2^. Comparisons of pathogenesis, viral load and clinical syndromes showed different responses to PRRSV infection among different pig breeds^3^,^4^,^5^,^6^,^7^. For example, Gottingen Miniature pigs had a virus load peaked on 12 dpi which is later than that in Pietrain pigs (6 dpi); moreover, the Miniature pigs had a smaller maximum virus load (10^2.5^ TCID_50_) than Pietrain pigs (10^4.5^ TCID_50_)^8^ Hampshire × Duroc cross bred pigs had slight change in weight, higher viremia and many lesions in lungs, whereas, NE Index line had bigger weight change, lower viremia and few lesions in lungs^9^. Importantly, serum viral load in Tongcheng pigs is significantly lower than that in Landrace pigs at 3, 5 and 7 dpi (data not shown). The results suggested that host genetic variation was associated with PRRS disease susceptibility^10^,^11^,^12^,^13^. However, the role of microRNA in immune responses to PRRSV infection of lung tissues from different pig breeds in *vivo* has not been explored previously.

MicroRNAs, with a length ranging from 21–24 nucleotides, are important post-transcriptional regulators playing critical roles in cell development^14^ and immune response^15^,^16^. Recent studies showed that microRNA affected replication of various viruses through binding to the genome of the viruses or regulating host antiviral pathways^17^,^18^,^19^,^20^. The potential role of microRNAs on PRRSV's replication and host immune pathways upon PRRSV infection has been reported. For example, miR-181 and miR-23a^21^,^22^ inhibited PRRSV replication through binding to PRRSV genome, while miR-181, miR-125b and miR-506^23^,^24^,^25^ suppressed PRRSV replication through regulating host antiviral pathways. Porcine alveolar macrophages (PAMs) are the target cells of PRRSV. Using high throughput sequencing technology, recent studies have identified 40 microRNAs involving in host immune response to PRRSV in PAMs^26^. However, microRNAs involved in regulating immune responses to highly pathogenic PRRSV infection *in vivo* need to be elucidated, e.g., by high throughput sequencing technology.

RNA editing is another important post-transcriptional modification that generates diversity between RNA and genomic DNA sequences. Evidence of RNA editing is evident in the transcripts of humans^27^,^28^, rhesus macaques^29^ and mice^30^. Adenosine (A) to inosine (I) conversion is the most prevalent type of RNA editing, which is catalyzed by ADAR (Adenosine deaminize acting on RNA) enzymes with double-stranded (ds) RNAs as substrates^31^. Accumulated data supports that microRNA editing plays prominent roles in many biological processes^32^,^33^,^34^,^35^, including virus-hosts interactions^36^. For example, RNA editing is increased due to the elevated expression of ADARs resulting in the Hepatitis Delta Virus replication inhibition^37^. Human cytomegalovirus (HCMV) infection induces the expression of ADAR1-p110, which regulates the editing of miR-376a^38^. In human cells, the Epstein-Barr virus (EBV) BART6 miRNA, which targets the Dicer nuclease that cleaves pre-miRNAs into miRNA duplexes, affects the latent state of EBV infection. A-to-I editing of the BART6 miRNA can suppress processing of pre-miRNAs to mature miRNAs by inhibiting Dicer cleavage in the cytoplasm^39^, and thus is potentially an important determinant in the regulation of EBV replication and latency^40^. Thus our interest is in the relationship between microRNA editing and PRRSV replication and host immune response to PRRSVs.

Herein, we identified microRNAs related to PRRSV replication and host immune responses using eight lung microRNA transcriptomes from Tongcheng or Landrace pigs infected with a highly pathogenic PRRSV. We identified the microRNAs that could bind to the PRRSV genome and candidate editing sites on microRNA sequences. Taken together, our results suggest critical and novel roles of microRNAs in the interactions between hosts and PRRSV *in vivo*.

## Results

### MicroRNA expression profiles of lung tissues after PRRSV infection

We generated eight microRNA expression profiles of lung tissues from Tongcheng or Landrace pigs infected with PRRSV (WUH3) at 3, 5 and 7 dpi and control individuals from the two breeds. The numbers of raw reads of these eight transcriptomes ranged from 18 to 30 million (Table 1). After filtering for reads with low quality and removing adaptor sequences contained in the raw reads (see Methods), the number of reads from these eight lung microRNA transcriptomes decreased to 14 to 25 million. Of the total 160 million clean reads, ~80% of them had a length of 21–24 nucleotides (Supplementary Figure S1). We then aligned them to the pig genome (Ensembl: Sscrofa10.2.71) using BWA (Burrows-Wheeler Aligner); approximately 56% to 86% mapped to the pig genome (Table 1). Unique mapped reads were used to annotate small RNAs against databases: Rfam-11.0, rnammer-1.2, snoRNA-LBME, GenomictRNAdatabase and miRBase-release19. Among the annotated small RNAs: rRNA, tRNA, snoRNA, Y_RNA and microRNA, more than 50% of the reads in each library were assigned as microRNA (Supplementary Figure S2). Detailed analyses suggested that 248 to 261 microRNAs were expressed in lung tissues of Tongcheng pigs, 249 to 261 microRNAs were expressed in lung tissues of Landrace pigs, and 239 microRNAs were transcribed in all the eight libraries. Combining results from all eight libraries, we obtained 278 known microRNAs. Moreover, we predicted novel microRNAs using mireap 2.0 software. Novel microRNA (Supplementary Table S1) prediction showed that 136 to 285 novel microRNAs were expressed in the lung tissues of Tongcheng pigs, 159 to 308 novel microRNAs were expressed in the lung tissues of Landrace pigs, while 294 novel microRNAs were transcribed in the combined eight libraries (Table 1).

### Identification of differentially expressed microRNAs after infection

**Figure 1:**
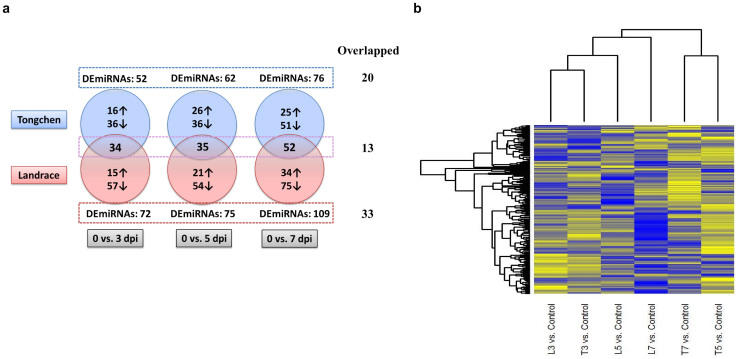
Differentially expressed microRNAs (DEmiRNAs) analysis. (a) Common DEmiRNAs among different time points and between the two breeds. (b) Heatmap for DEmiRNAs at 3, 5, 7 dpi compared with control groups in the two breeds. The log_2_ fold changes were used to plot the heatmap. MicroRNAs shown in yellow had up-regulated expression and those shown in blue had down-regulated expression in the lungs of infected individuals compared with control groups. Hierarchical clusters of microRNAs and samples were clustered using default set of heatmap.2.

The relative expression levels of microRNAs were normalized as TMM (Trimmed Mean of M-values) using edgeR package. Compared with control groups, we defined microRNAs with a fold change (FC) > 2 and false discovery rate (FDR) < 0.05 as significantly differential expressed microRNAs (DEmiRNAs). For simplicity, compared with control groups, we referred microRNAs having significantly differential expression in PRRSV-infected Tongcheng pigs at 3, 5 and 7 dpi as set 1, set 2 and set 3, respectively. We merged DEmiRNAs of set 1, set 2 and set 3 into set 4. Similarly, we referred DEmiRNAs in PRRSV-infected Landrace pigs at 3, 5 and 7 dpi compared with control animals of this breed as set 5, set 6 and set 7, respectively. We also merged DEmiRNAs of set 5, set 6 and set 7 into set 8. Detailed analysis on the up-/down-regulated DEmiRNAs showed that the amounts of down-regulated DEmiRNAs were more than the amounts of up-regulated in all the six sets (set 1: 36 (down) vs 16 (up); set 2: 36 (down) vs 26 (up); set 3: 51 (down) vs 25 (up); set 5: 57 (down) vs 15 (up); set 6: 54 (down) vs 21 (up); set 7: 75 (down) vs 34 (up)) (Fig. 1a), which indicated that down-regulation of DEmiRNAs might be a common strategy for pig responses to HP-PRRSV (High Pathogenic-PRRSV) infection. After that, the top 10 most up-/down-regulated microRNAs at 3, 5, 7 dpi were identified compared with control group (Supplementary Table S2). Ssc-miR-183 was the most up-regulated microRNA consistently at 3 dpi (log_2_Fold Change (FC) = 4.93), 5 dpi (log_2_FC = 5.33) and 7 dpi (log_2_FC = 2.68) in Landraces' lungs. Ssc-miR-215 was the most down-regulated microRNA at 3 dpi (log_2_FC = -5.58), 5 dpi (log_2_FC = -7.36) and ssc-miR-122 was the most down-regulated microRNA at 7 dpi (log_2_FC = -5.71) in Landraces' lungs. While, in the lungs of Tongcheng pigs, the most up-regulated microRNAs at 3, 5 and 7 dpi were ssc-miR-490-5p (log_2_FC = 2.15), ssc-miR-183 (log_2_FC = 2.8) and ssc-miR-215 (log_2_FC = 6.2), respectively. Correspondingly, the most down-regulated microRNAs were ssc-miR-4332 (log_2_FC = -4.19), ssc-miR-374b-3p (log_2_FC = -6.07) and ssc-miR-95 (log_2_FC = -2.96).

The numbers of DEmiRNAs in set1, set2, set3 and set4 were 52, 62, 76 and 116. Detailed analysis showed that 20 DEmiRNAs were common in all set1, set2 and set3, suggesting that these DEmiRNAs might play roles in persistent PRRSV infection in the lungs of Tongcheng pigs (Fig. 1a). The numbers of DEmiRNAs in set 5, set 6, set 7 and set 8 were 72, 75, 109 and 153, which was more than that in Tongcheng pigs correspondingly. A total of 33 microRNAs (Table 2) has significantly differential expression in sets5, 6, and 7, which was more than that in Tongcheng pigs (20 DEmiRNAs). There were 13 DEmiRNAs were differentially expressed at all the three time points in both pig breeds. Among the 13 DEmiRNAs, only four microRNAs: miR-143-3p, miR-183, miR-219 and miR-28-3p were up-regulated and the other nine microRNAs were all down-regulated in both breeds (Table 2) at all the time points. This result indicated that these microRNAs might be co-regulated during PRRSV infection in both breeds. Next, we merged set 4 and set 8 to obtain 174 DEmiRNAs. The heatmap for these 174 microRNAs' Log_2_FC revealed that expression patterns of DEmiRNAs in Tongcheng pigs at 3 dpi clustered with those of Landrace pigs at 3 dpi, whereas the DEmiRNAs expression patterns at 5 and 7 dpi were clustered within each breed (Fig. 1b). The dynamic changes of microRNA expression revealed that the two pig breeds had similar microRNA expression pattern at 3 dpi, with different microRNA expression pattern at 5 and 7 dpi. Furthermore, we defined Tongcheng specific DEmiRNAs as the miRNAs differentially expressed significantly at all-time points (3, 5 and 7 dpi) in Tongcheng pigs, but not in Landrace pigs, and vice versa for Landrace specific DEmiRNAs. Next, we identified seven Tongcheng specific DEmiRNAs and 20 Landrace specific DEmiRNAs. Among the seven Tongcheng specific DEmiRNAs, ssc-miR-204 was the most abundant expressed (Table 3). Specifically, compared with control group, miR-22-5p was significantly up-regulated at 3 dpi (Log_2_FC = 1.14, FDR = 1.19e-42), 5 dpi (Log_2_FC = 1.57, FDR = 1.23e-91), and 7 dpi (Log_2_FC = 1.11, FDR = 1.57e-39) in the lungs of Tongcheng pigs, while not altered significantly in the lungs of Landrace pigs at all the three comparisons. These findings indicated that miR-22-5p might participate in specific responses to PRRSV infection in Tongcheng pigs. Among the 20 specific DEmiRNAs in Landraces' lungs, six DEmiRNAs (ssc-let-7i, ssc-miR-122, ssc-miR-195, ssc-miR-146b, ssc-miR-146a-5p, ssc-miR-30b-5p) had an expression value (TMM) larger than 10,000 at 0 dpi and all of them were down-regulated significantly at 3, 5, 7 dpi (Table 3). These results implied that these six DEmiRNAs might contribute to the Landrace specific responses to PRRSV infection.

**Figure 2:**
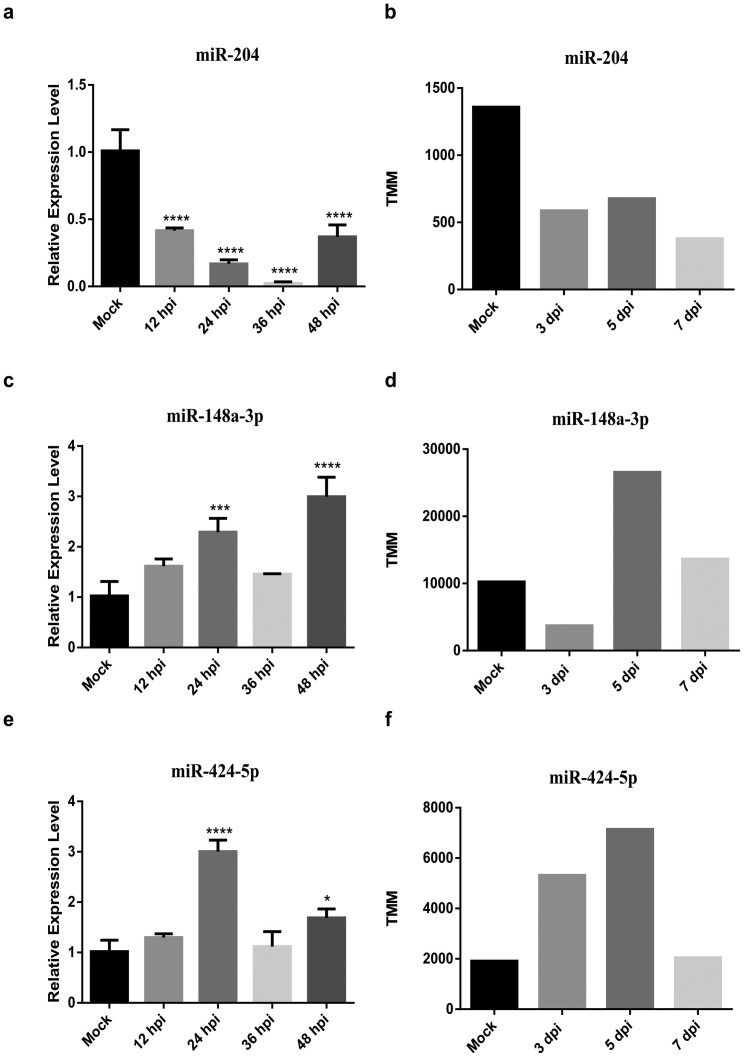
Validation of DEmiRNAs in PAMs from the lung of Tongcheng pig using qRT-PCR. We randomly chose three DEmiRNAs (miR-204, miR-148a-3p, miR-424-5p) in the lung tissues of Tongcheng pigs from our data. (a), (c) and (e) are the qRT-PCR results; (b), (d) and (f) are TMM values of microRNAs. All the data for qRT-PCR were normalized to U6 and were represented as mean ± SD (n = 3). Statistical significance was analyzed by ANOVA; *p < 0.05; **p < 0.01; ***p < 0.001; ****p < 0.0001.

**Figure 3:**
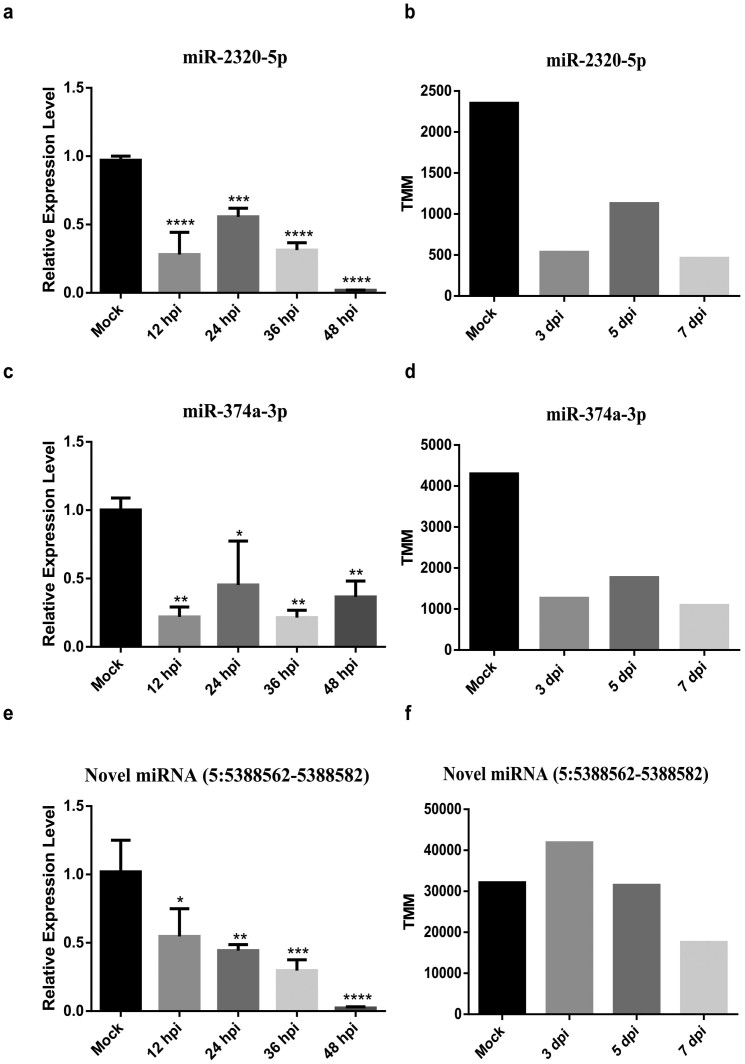
Validation of DEmiRNAs in PAMs from the lung of Landrace pig using qRT-PCR. We randomly chose three DEmiRNAs (miR-2320-5p, miR-374a-3p, Novel miRNA (5:5388562-5388582)) in the lung tissues of Landrace pigs from our data. (a), (c) and (e) are the qRT-PCR results; (b), (d) and (f) are TMM values of microRNAs. All the data for qRT-PCR were normalized to U6 and were represented as mean ± SD (n = 3). Statistical significance was analyzed by ANOVA; *p < 0.05; **p < 0.01; ***p < 0.001; ****p < 0.0001.

We also performed qRT-PCR experiments to validate several DEmiRNAs in mock and infected PAMs (Pulmonary Alveolar Macrophages) from the lungs of Tongcheng and Landrace pig. We examined the microRNA expression level in mock PAMs and infected PAMs with PRRSV WUH3 (MOI = 0.2) at 12, 24, 36 and 48 hpi (hour post infection). The qRT-PCR results showed that the expression patterns of miR-204 and miR-424-5p in the PAMs of Tongcheng pigs after infection with PRRSV WUH3 are similar with those obtained from our deep sequencing data analysis (Fig. 2a, b, e and f). The miR-148a-3p was down-regulated at 3 dpi in our data, but the qRT-PCR results showed that it was up-regulated at all the time point we detected (Fig. 2c, d). High consistency of expression patterns of miRNA-2320-5p and miR-374a-3p were observed between qRT-PCR and the deep sequencing results in Landrace pigs (Fig. 3a, b, c and d). The expression pattern of novel miRNA (5: 5388562-5388582) detected by qRT-PCR was similar with the deep sequencing results though it was up-regulated at 3 dpi in the deep sequencing (Fig. 3e, f). Overall, the six DEmiRNAs we detected all showed similar expression pattern compared with the small RNA sequencing results, which indicated that our analysis results were highly reliable.

### Functional annotation of the target genes of specific DEmiRNAs

We predicted target genes of specific DEmiRNAs in Tongcheng (seven DEmiRNAs) and Landrace (20 DEmiRNAs) pigs using the experimental validated microRNA-targets databases: miRecord (version4), mirTarBase 4.5, TarBaseV5.0 and mirTarPri. This resulted in 78 predicted targets for three out of seven Tongcheng specific DEmiRNAs and 1154 targets for nine out of 20 Landrace specific DEmiRNAs. Gene Ontology (GO) analysis showed that the target genes of DEmiRNAs specific in Tongcheng pigs significantly enriched in regulation of transcription, DNA dependent (FDR = 0.018548), T cell differentiation (FDR = 0.018548), regulation of cell cycle process (FDR = 0.027894) (Supplementary Table S3). These results showed that these DEmiRNAs mainly regulate immune related responses and transcriptional activities. In contrast, the target genes of Landrace specific DEmiRNAs were enriched in G protein-coupled receptor signaling pathway (FDR = 9.94e-26) and signal transduction (FDR = 1.89e-06).

**Figure 4:**
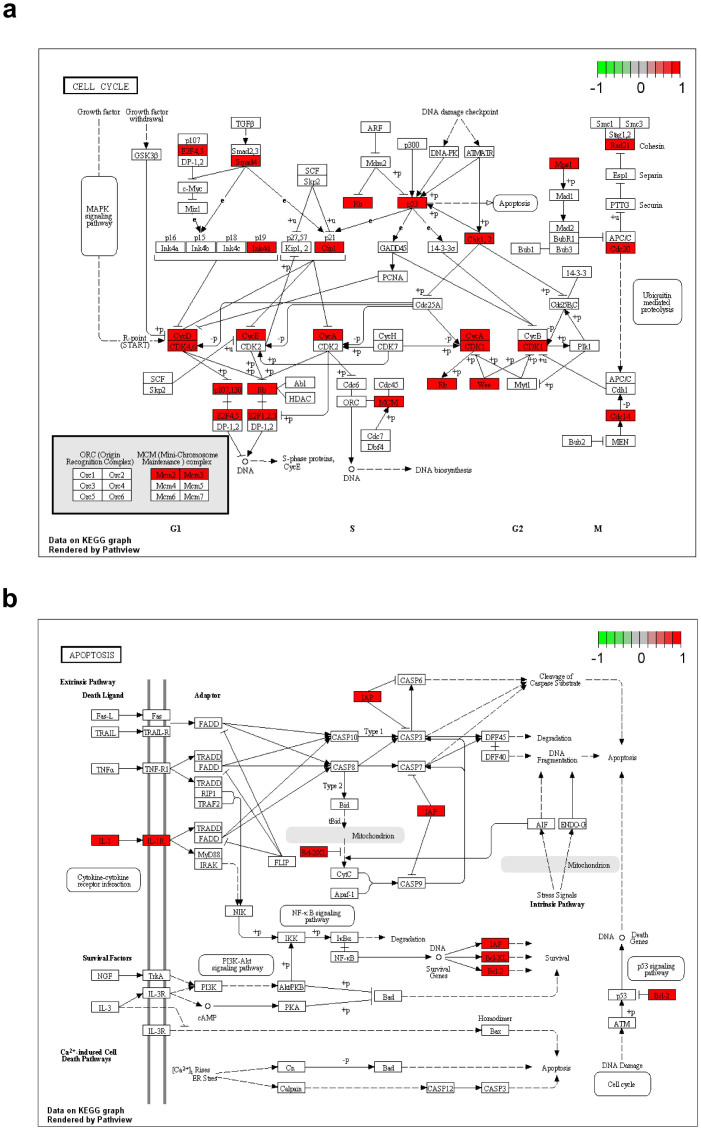
Target genes of Tongcheng/Landrace specific DEmiRNAs' KEGG pathways enrichment analysis (a) Landrace specific DEmiRNAs target genes enriched in cell cycle pathway. (b) Tongcheng specific DEmiRNA target genes enriched in apoptosis pathway. The red boxes represent the target genes of Tongcheng or Landrace specific DEmiRNAs.

KEGG pathway enrichment analysis showed that Tongcheng specific DEmiRNAs' targets were significantly (p < 0.05) enriched in 24 pathways, while Landrace specific DEmiRNAs' targets were enriched in 38 pathways (Supplementary Table S4). Among the 38 pathways, cell cycle pathway (p = 7.44e-08) (Fig. 4a) was enriched in Landrace pigs consistent with the GO analysis. However, the Tongcheng specific DEmiRNAs' targets were not enriched significantly in cell cycle pathway. These findings indicated that cell cycle related pathways were differentially regulated after infection with PRRSV in Landrace pig lungs. Apoptosis is an important defense mechanism to virus infection. The targets of Tongcheng, but not Landrace, specific DEmiRNAs were significantly enriched in apoptosis pathway (p = 0.008321) (Fig. 4b). Additionally, the targets of Landrace specific DEmiRNAs, but not Tongcheng, were enriched significantly in Toll-like receptor signaling pathway (p = 4.59e-05) and RIG-I-like receptor signaling pathway (p = 0.001425) (Supplementary Table S4). Above all, these results indicated that the specific DEmiRNAs of Tongcheng or Landrace pigs represent different pathways activated by PRRSV infection.

### Prediction of microRNA binding sites on the conserved regions of PRRSV genome

**Figure 5:**
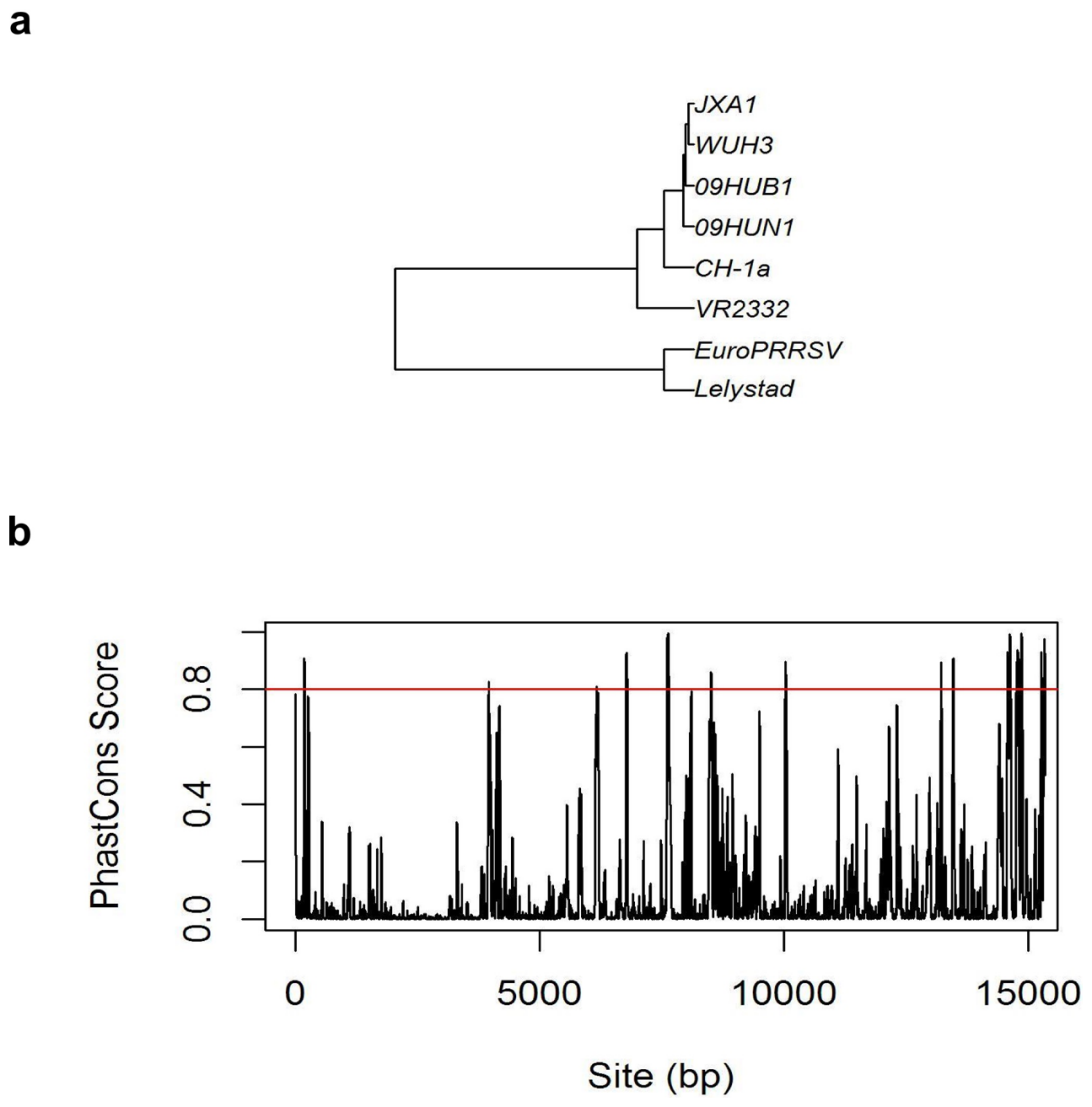
Comparison of eight strains of PRRSV using evolutionary analysis and PhastCons Score distribution along WUH3 genome (a) The evolutionary relationship among the eight strains. (b) The distribution of PhastCons Score for each base on WUH3 genome. Red line represents the threshold (PhastCons Score = 0.8) for conservation.

Using miRanda v3.3a software, we predicted 243 cellular microRNAs that can bind to WUH3 PRRSV genome (downloaded from http://www.ncbi.nlm.nih.gov/, accession number: HM853673.2). Among them, six microRNAs (ssc-miR-128, ssc-miR-186, ssc-miR-4332, ssc-miR-218-3p, ssc-miR-2320-5p and ssc-miR-150) were predicted to bind to the 3′UTR of WUH3 PRRSV genome. As the conserved sequences among different strains of viruses might play critical roles on their pathogenicity, the microRNA that can bind to the conserved sequences among different PRRSV strains' genome might be important to the control of the viruses' pathogenicity. Therefore, we downloaded seven other PRRSV strain genome sequences from NCBI (Fig. 5a and Supplementary Table S5). The sequence conservation can be measured by PhastCons value^41^. We first aligned the eight strains of PRRSV genome sequences. Then, the PhastCons Score for each base of the genome of WUH3 strain was calculated (Method) (Fig. 5b). The continuous bases (length > 20 bp) with PhastCons Score larger than 0.8 were considered as conserved sequences among the eight strains of PRRSV. If we set the threshold of PhastCons score as 0.7, miR-181 can be predicted to bind to the conserved region, as confirmed previously^19^. We identified 15 conserved regions among the eight PRRSV strains. A total of 14 microRNAs were predicted to bind to these conserved regions of WUH3 PRRSV genome. Among them, miR-2320-5p can bind to 14847 bp–14869 bp on WUH3 PRRSV genome which is close to the 3′UTR (15171 bp–15347 bp) (Supplementary Table S6). Meanwhile, miR-2320-5p also can bind to the 3′UTR (15226 bp–15248 bp) of WUH3 PRRSV genome. In both breeds, miR-2320-5p was down-regulated significantly at 3 dpi (Tongcheng: log_2_FC = -2.20, p = 7.147e-144, FDR = 3.140e-143; Landrace: log_2_FC = -2.14, p = 1.132e-269, FDR = 4.758e-269) compared to control. Taken together, we speculated that the expression of miR-2320-5p might be modulated by PRRSV infection due to the potential combination with PRRSV genome.

### Putative microRNA editing during the PRRSV infection

**Figure 6:**
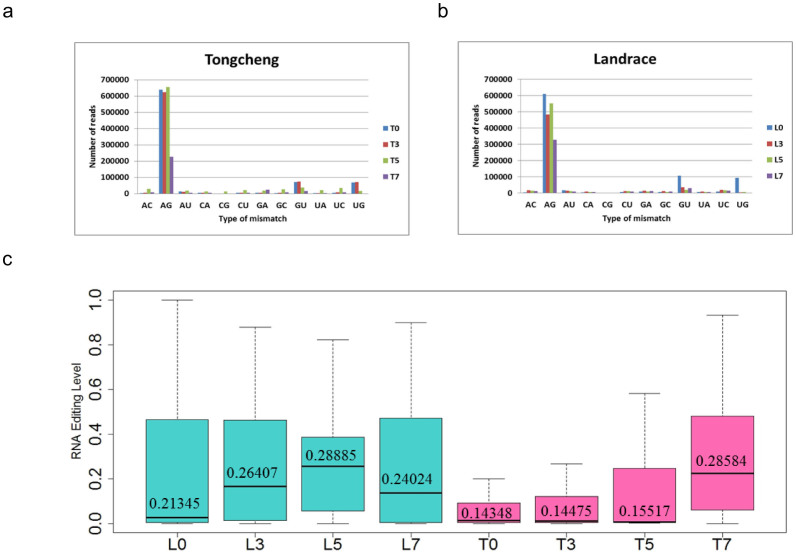
The microRNA editing profile during the HP-PRRSV infection in the lungs of Tongcheng and Landrace. (a), (b) Distribution of the number of editing type in the lungs of the two breeds. (c) Average microRNA editing level in control groups (L0, T0) and after infection in the two breeds. The RNA editing level was showed as the ratio of the reads support the mismatch in the site to the total reads detected at this site. The statistic method used in Figure 6c is Kolmogorov-Smirnov test (K-S test). L0 vs. L3: p-value = 0.006148; L0 vs. L5: p-value = 0.000225; L0 vs. L7: p-value = 0.1272; T0 vs. T3: p-value = 0.9997; T0 vs. T5: p-value = 0.07608; T0 vs. T7: p = 4.884e-07.

**Figure 7:**
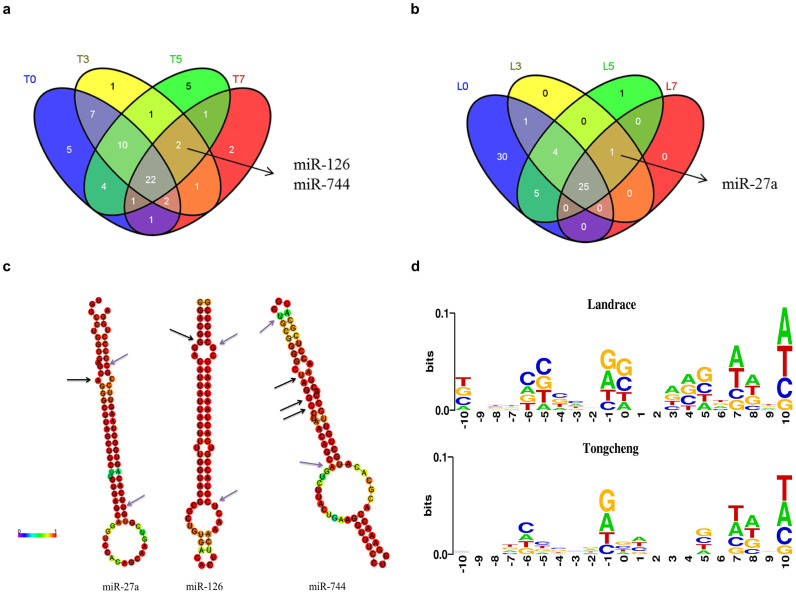
Common and specific microRNAs undertaken microRNA editing (a),(b) Venn diagram showing the common and specific microRNAs undertook editing in the four time points in the two breeds. (c) Second structure and the editing sites of microRNAs induced by PRRSV infection in the two breeds. The black arrow indicated RNA editing site; The sequence between the purple arrows indicated the mature sequence. (d) Sequence motif for the 10 bp up-/downstream of the editing site.

We identified candidate microRNA editing sites and editing level (Supplementary Table S7) in the lungs from control and infected pigs using previously described methods^42^. A total of 150 and 168 microRNA editing sites (corresponding to 67 and 65 microRNAs) were identified in the lung tissues of Landrace and Tongcheng pigs, respectively (Supplementary Table S8) with 21 microRNAs overlapping. The A-G conversion was the most prevalent among all the eight libraries (Fig. 6a, b). Moreover, we used WebLogo 3.4 to characterize the sequence motif of 10 bp up-/down-stream of the microRNA editing site. In both breeds, we found that at -1 site, G and A were the most prevalent bases and at +10 site, T and A were the most prevalent bases (Fig. 6d). MicroRNA editing level was defined as the ratio of the number of reads supporting the mismatch at the site to the total number of reads detected at this site (Methods). Interestingly, the average RNA editing level in Tongchengs' lungs gradually increased (T0: 0.14248; T3: 0.14475; T5: 0.15517; T7: 0.28584) during to the time-course of HP-PRRSV infection. In Landrace's lungs, the average editing level increased from 0 dpi to 5 dpi (L0: 0.21345; L3: 0.26407; L5: 0.28885) and decreased at 7 dpi (L7: 0.24024) compared with 5 dpi, but still higher than 0 dpi (Fig. 6c). Importantly, compared with the controls (0 dpi), the average RNA editing level in microRNAs were significantly increased at 7 dpi (p = 4.88e-07) in Tongchengs' lungs, and were significantly increased at 3 dpi (p = 0.0061) and 5 dpi (p = 0.00025) in Landraces' lungs. These findings suggested that infection of WUH3 PRRSV caused a significant increase in average microRNA editing level in lung tissues. Next, we identified 22 and 25 microRNAs that were edited at all the time points in the lungs of Tongcheng and Landrace, respectively (Fig. 7a,b). Previous studies implicated that microRNA editing might occur during virus infection^34^,^35^,^36^,^37^,^38^. We found almost 20% (13 out of 65) microRNA were edited after infection in lungs of Tongcheng pigs; this was larger than the corresponding ratio 3% (2 out of 67) in lungs of Landrace pigs. Two out of 13 microRNAs (miR-126 and miR-744) were edited in the lungs of Tongcheng at all the three time points (3, 5, 7 dpi) and one microRNA (miR-27a) was edited in the lungs of Landrace at 3, 5, 7 dpi (Fig. 7c). More importantly, miR-181 was also found edited (Supplementary Table S7). Above all, our findings indicated increased microRNA editing levels in PRRSV infected lungs in both pig breeds.

## Discussion

To investigate the roles of microRNAs in interplay between hosts and PRRSV *in vivo*, we characterized microRNA profiles in lung tissues of pigs (Tongcheng pigs and Landrace pigs) infected with the WUH3 PRRSV (3, 5, 7 dpi) and the lung tissues from control groups of these two breeds. We found 278 known microRNAs expressed in the combined eight microRNA libraries. Among these, ~50% (116 DEmiRNAs in Tongcheng group; 153 DEmiRNAs in Landrace group) were altered significantly after PRRSV infection compared with control groups. The large ratio of DEmiRNAs in total microRNAs suggested that microRNAs play important roles during PRRSV infection in the lung tissues of both breeds. KEGG and GO analysis of target genes of specific DEmiRNAs revealed that different pathways were activated in the two breeds. We predicted 14 microRNAs that can bind to the conserved sequences of eight PRRSV strains (Fig. 5a, Supplementary Table S5). Moreover, we identified candidate microRNA editing sites of microRNAs in the PRRSV infection process. Compared with control groups, the microRNA editing level changed significantly after inoculation with PRRSV, indicating microRNA editing was involved in interactions between hosts and PRRSV.

Among the 13 DEmiRNAs that significantly differentially expressed at all the three time points in both breeds, miR-144, miR-219 and miR-374a were also differentially expressed in PAMs after infection with PRRSV^24^, and miR-183, miR-219, miR-28-3p and miR-143-3p were all up-regulated significantly at 3, 5, 7 dpi in both breeds. Specifically, previous studies showed that miR-143 could reduce cell proliferation and induce apoptosis through down-regulating DNMT3A^43^ in human cells. And, PRRSV infection can result in apoptosis of host cells^44^, implying that PRRSV infection might lead to cell apoptosis through up-regulating the expression level of miR-143. These results suggested that these microRNAs might be co-regulated and play critical roles in immune responses to PRRSV infection in lung tissues from the two breeds.

Tongcheng specific or Landrace specific DEmiRNAs might reflect breed specific anti-virus mechanisms. Compared with control groups, Tongcheng specific DEmiRNA-22-5p was significantly up-regulated at all the time points. MiR-22 was reported that it could significantly suppress the activity of NF-kB by regulating the expression of nuclear receptor coactivator 1 (NCOA1)^45^. The up-regulation of miR-22-5p might be a strategy for virus to suppress hosts' immune responses in Tongcheng groups, but not in Landrace groups. MiR-122 and miR-215, as Landrace specific DEmiRNAs, were both significantly down-regulated at 3, 5 and 7 dpi. Previous studies showed that miR-122 and miR-215^46^ were able to enhance the replication of HCV. Down-regulation of both microRNAs might be an anti-viral strategy for Landrace pigs.

Modulating host cell cycle is a common strategy used by viruses to favor their extremely fast propagation. For example, it has been reported that HIV-1, HSV-1, CMV and EBV^47^,^48^,^49^ arrest human cells in different phases. GO analysis for targets of specific DEmiRNAs in Tongchengs are enriched in one cluster related to DNA damage response (FDR = 0.0185), indicating that the genes involved in the DNA damage responses which mainly occurred in G2 phase were deregulated. Landrace specific DEmiRNA, miR-122 was down-regulated ~ 2 fold at 3 dpi and ~ 10 and ~ 15 fold at 5 and 7 dpi. CDK4, an important regulator of cell cycle, is the target of miR-122^50^. This result indicated that miRNA-122 might perturb the normal cell progression by regulating the expression level of CDK4. What's more, Marc-145 cells could be arrested in S phase to favor PRRSV replication^51^ consistent with the microarray analysis of PAMs' transcriptional responses to HP-PRRSV (WUH3)^52^. Moreover, the KEGG analysis showed that the targets of Landrace specific DEmiRNAs were enriched in the cell cycle pathway (p = 7.44e-08). Taken together, the data supports our hypothesis that altering the expression level of microRNAs induced by HP-PRRSV infection could modulate the cell cycle.

Our results showed that A to G conversion is the most prevalent mismatch type (Fig. 7a, b), which is consistent with that in human transcriptomes^26^. Previously studies showed that ADAR1 transcription both in human and mouse is IFN inducible^53^,^54^ with the editing level positively correlated with the expression level of ADAR in different tissues^27^. It is possible that PRRSV infection results in up-regulation of expression of ADAR leading to elevated RNA editing after infection. In our study, after infection, the average microRNA editing levels were higher than control groups in both breeds (Fig. 7c). Growing evidence suggests that microRNA editing might involve immune responses to virus infection. During HCMV infection, edited miR-376 down-regulates the expression of HLA-E which promotes NK cell ability to eliminate the HCMV infected cells^60^. Further study showed that microRNA editing occurred in pre-miRNA-126, pre-miRNA-744 in Tongcheng and in pre-miRNA-27a in Landrace at 3, 5 and 7 dpi (Supplementary Table S5). It has been demonstrated that microRNA editing can dramatically change target specificity^33^,^55^,^56^ Compared with control microRNAs^30^. Although, the editing sites in miR-126, miR-744 and miR-27a were not in conserved seed sequences, they might affect the processing of mature microRNAs by down-regulating their expression levels. A previous study showed that miR-27a can keep inflammation from over responding^57^. Compared with control groups, miR-27a was down-regulated at 3 dpi (log_2_FC = -0.75), 5 dpi (log_2_FC = -0.64) and 7 dpi (log_2_FC = -0.26), in Landrace pigs possibly due to microRNA editing altering immune responses to PRRSV infection. Compared with control group, miR-744 was ~15% down-regulation in the lungs of Tongcheng pigs at 3 dpi; conversely, it was ~18% up-regulated in the lungs of Landrace pigs at 3 dpi. Previous studies showed that TGF-β1 is the direct target of miR-744^58^. It is possible that down-regulated miR-744 might up-regulate the expression of regulator of B-cell homeostasis, TGF-β, which might contribute to the elimination of PRRSV^59^.

In summary, comparing the microRNA expression profiles between Tongcheng and Landrace pigs after PRRSV infection indicated that pathways were differentially regulated between the two pig breeds. These findings provide insights to the mechanisms about how genetic factors influence the anti-viral strategy through regulating microRNA expression. We first predicted potential microRNA editing events during PRRSV infection; compared with control groups, the significant increase of RNA editing level after infection needs further functional validation. Above all, our results provided novel and crucial roles of microRNAs on PRRSV infection *in vivo* and provide insights into the mechanisms of PRRSV pathogenicity.

## Methods

### Ethics statement

All experiments were performed in accordance with relevant guidelines and regulations; the Animal Care and Use Committee of Huazhong Agricultural University approved this research. The samples collection procedures according with the Guidelines of Huazhong Agricultural University, and scientific, ethical and legal principles of the Hubei Regulations for the Administration of Affairs Concerning Experimental Animals which have been accepted world-widely. Animal treatment is in strict accordance with the 《Recommendations in the Hubei Regulations for the Administration of Affairs Concerning Experimental Animals, 2005》, which has been approved by the Department of Science and Technology of Hubei Province in China (Permit Number: SYXK(ER)2010-0029).

### Animal challenge, samples collection and small RNA sequencing

A total of 24, 5 weeks old pigs (12 Tongcheng pigs and 12 Landrace pigs), free from PRRSV, were randomly chosen to perform further experiments. Three Tongcheng pigs and three Landrace pigs were randomly selected as control groups. The remaining 18 pigs were inoculated with a high pathogenic PRRSV (WUH3) in muscle at a 3 mL 10^5^ TCID_50_ dose for each pig. At 3, 5, 7 dpi, we randomly slaughtered three Tongcheng pigs and three Landrace pigs. The lung tissues from the slaughtered pigs were collected and were immediately snap-frozen in liquid nitrogen and stored at −80°C until use. The total RNA from each lung was extracted using Trizol reagent (Invitrogen, Carlsbad, USA) following the manufacture's recommendations. RNA quality and quantity were evaluated using Agilent 2100 Bioanalyzer. Equal quantities of RNA isolated from three lungs from three pigs at each time point were pooled. Totally, eight small RNA libraries were constructed according to the standard illumina protocols and were sequenced on solexa platform. The deep sequencing data have been deposited in NCBI SRA database and are accessible through GEO series accession number GSE58436.

The PAMs were obtained by lavaging lungs of 8-weeks-old Landrace and Tongcheng pigs then cultured in RPMI 1640 medium supplemented with 10% FBS and penicillin/streptomycin. Cells were harvested using EASYspin kit produced by Aidlab Biotechnologies Co., Ltd and RNA was extracted following the manufacturer's protocol. U6 RNA was set up as endogenous control. M-MLV Reverse Transcriptase (Takara) was used to reverse transcribes miRNAs. The qRT-PCR was performed in LightCycler® 480 Real-Time PCR System. The primers used for qRT-PCR were listed in Supplementary Table S9.

### Alignment and annotation of small RNAs

The raw reads were filtered by following steps: (1). Trimmed the bases with low quality (<20) from 5′ end; (2). Discarded the whole reads with average quality less than 20; (3). Clipped adaptor sequence and discarded reads shorter than 18 bp; then, the clean reads were obtained. We used BWA (default set) to align the clean reads on the pig genome (Sscrofa10.2.71). Only the aligned reads mapping uniquely were used for further analysis. Then, all the reads were compared with pig mature microRNA sequences (miRBase release 19) using blastn. The parameters were set to 95% identity and 80% coverage. Blastn and perl scripts were used to calculate the reads count for each microRNA. Novel microRNAs were predicted using mireap 2.0 software. The edgeR package was used to normalize the expression value of microRNAs (TMM) in different libraries and call differential expressed microRNAs (fold change > 2 and FDR < 0.05). We set the dispersion as 0.1 according to previous study^61^. The false discovery rate was calculated using Benjamini algorithm.

### Target prediction and functional analysis

The targets of microRNAs were extracted from experimental validated miRNA-mRNA databases: miRecord version4, mirTarBase 4.5, TarBaseV5.0 and mirTarPri. Functional annotation of predicted microRNA targets were performed based on Gene Ontology (GO) database and the pathways that they enriched in were analyzed using Kyoto Encyclopedia of Genes and Genomes database (KEGG). The significantly enriched functional categories were determined by the multiple test adjustment proposed by Benjamini & Hochberg. KEGG pathway enrichment analysis and visualization were performed using R packages: GOstats and pathview. The KEGG pathways with a p < 0.01 were defined as significantly enriched pathways.

### Prediction of microRNA binding sites on PRRSV genome

We downloaded the genome of WUH3 strain (NCBI accession number: HM853673.2) of PRRSV from NCBI (http://www.ncbi.nlm.nih.gov/). The microRNAs binding sites on PRRSV genome were predicted using miRanda v3.3a software. And the coordinates of microRNAs bind sites on PRRSV(WUH3) genome were extracted using perl scripts. We downloaded other seven strains PRRSV's genome sequences from NCBI (Fig. 5a). To identify the conserved regions among the eight strains, the PhastCons scores for each base were calculated (Fig. 5b) by the following steps: first, the muscle 3.8.31 software was used to create alignment fasta documents of PRRSV genomes. And the evolutionary tree was plotted according to the fasta document using FigTree. Second, we used phyloFit to create *.mod file, which was the input file for phastCons socre calculation; third, phastCons score, was calculated by using phastCons software. Perl scripts were used to extract the consecutive regions' with average phastCons score larger than 0.8. We used bedtools intersect function to get the microRNAs that were predicted to bind to the conserved sites on WUH3 genome.

### Identification of potential microRNA editing sites

According to the method described in Ref. 37 (detailed in Supplementary), we identified microRNA editing sites located on the known microRNA sequences. The common and specific microRNAs undertook RNA editing across all the time points in the lungs of Tongcheng and Landrace were analyzed using perl scripts. The RNA editing level was showed as the ratio of the reads support the mismatch in the site to the total reads detected on this site. The significance of the pairwise comparisons of average microRNA editing level between control group and infected groups in the two breeds was evaluated using Kolmogorov-Smirnov test. The sequence motif of 10 bp up/down stream around the microRNA editing site in the lungs of Tongcheng and Landrace pigs were analyzed using WebLogo 3.4.

## Supplementary Material

Supplementary Information Supplementary Information

## Figures and Tables

**Table 1:** Sequencing data filter and mapping

	L0 (Control)	L3 (3 dpi)	L5 (5 dpi)	L7 (7 dpi)	T0 (Control)	T3 (3 dpi)	T5 (5 dpi)	T7 (7 dpi)
Raw reads	18455183	19359113	28886943	30282221	18121314	19868517	27941576	28358232
Clean reads	16497848	16457860	25964127	21508237	16501725	14276535	23607746	17275229
Mapped reads	15166411 (82.18%)	15918810 (82.23%)	25082301 (86.83%)	20518759 (67.76%)	15355797 (84.74%)	13735202 (69.13%)	22799233 (81.60%)	15999510 (56.42%)
Unique mapped reads	7749540	8035638	12888438	11029048	8762889	6673943	12018715	8927363
Detected Known miRNAs (reads > 1)	255	249	261	250	249	249	261	248
Novel miRNAs (reads > 1)	159	178	308	239	136	182	285	183

Note: T: Tongcheng; L: Landrace. dpi: day post infection.

**Table 2:** Significantly differential expressed microRNAs at all the time points (3,5,7 dpi) compared with control individuals in the two breeds

Landrace	microRNA	0 dpi	3 dpi	5 dpi	7 dpi
		TMM			
Up	**ssc-miR-183**	65	1961	2588	412
	ssc-miR-30c-3p	2610	10670	6738	7063
	**ssc-miR-143-3p**	192555	578719	1146757	974013
	**ssc-miR-28-3p**	541	1274	1301	1455
	**ssc-miR-219**	40	84	112	131
	ssc-miR-129a	639	1331	307	89
Down	ssc-miR-215	3863	81	24	1268
	ssc-miR-4332	235	20	59	55
	ssc-miR-382	37	7	9	11
	**ssc-miR-144**	193	34	42	90
	ssc-let-7i	580833	105860	115555	279454
	ssc-miR-2320-5p	2350	534	1128	462
	ssc-miR-31	9513	2276	3576	1154
	ssc-miR-301	97	24	26	25
	**ssc-miR-1839-3p**	65	16	9	6
	**ssc-miR-374a-3p**	4298	1265	1768	1088
	ssc-miR-432-5p	161	48	20	27
	**ssc-miR-95**	518	175	72	85
	ssc-miR-345-3p	2181	742	974	776
	ssc-miR-452	178	65	34	73
	ssc-miR-664-5p	1553	613	741	447
	ssc-miR-146a-5p	13012	5145	2046	1704
	ssc-miR-146b	13667	5405	2078	1732
	**ssc-miR-145-3p**	390	159	98	170
	**ssc-miR-142-5p**	8382	3461	3670	932
	ssc-miR-205	911	379	305	179
	**ssc-miR-374a-5p**	7033	2934	3216	1330
	**ssc-miR-126-5p**	13575	5762	2546	6759
	**ssc-miR-374b-5p**	6084	2613	2791	723
	ssc-miR-195	26235	11589	12185	2585
	ssc-miR-628	128	62	30	11
	ssc-miR-30b-5p	11423	5569	4200	2780
	ssc-miR-122	55623	27245	5801	1063

Tongcheng	microRNA	0 dpi	3 dpi	5 dpi	7 dpi
		TMM			
Up	**ssc-miR-183**	87	270	605	364
	**ssc-miR-219**	31	91	97	99
	**ssc-miR-143-3p**	119229	322185	460651	626459
	ssc-miR-491	43	108	103	94
	ssc-miR-28-3p	473	1105	1223	1604
	ssc-miR-22-5p	406	897	1206	874
	ssc-miR-345-5p	52	108	191	103
Down	**ssc-miR-144**	92	13	46	24
	**ssc-miR-1839-3p**	56	12	2	10
	ssc-miR-95	681	140	208	88
	ssc-miR-374b-3p	864	242	13	277
	ssc-miR-450b-5p	49	16	8	13
	**ssc-miR-145-3p**	322	106	52	129
	ssc-miR-374a-3p	3076	1056	794	527
	**ssc-miR-142-5p**	12035	4481	966	4387
	ssc-miR-10a-3p	186	75	67	59
	ssc-miR-374b-5p	4159	1745	1180	881
	ssc-miR-204	1359	588	678	380
	ssc-miR-374a-5p	4881	2213	1433	1026
	**ssc-miR-126-5p**	8267	4018	2490	3697

Note: the “bold” means overlapped DEmiRNAs between the two breeds

**Table 3:** Tongcheng/Landrace specific DEmiRNAs compared with control individuals

Tongcheng	microRNA	0 dpi	3 dpi	5 dpi	7 dpi
		TMM			
	ssc-miR-204	1359	588	678	380
	ssc-miR-374b-3p	864	242	13	277
	ssc-miR-22-5p	406	897	1206	874
	ssc-miR-10a-3p	186	75	67	59
	ssc-miR-345-5p	52	108	191	103
	ssc-miR-450b-5p	49	16	8	13
	ssc-miR-491	43	108	103	94

Landrace	microRNA	0 dpi	3 dpi	5 dpi	7 dpi
		TMM			
	ssc-let-7i	580833	105860	115555	279454
	ssc-miR-122	55623	27245	5801	1063
	ssc-miR-195	26235	11589	12185	2585
	ssc-miR-146b	13667	5405	2078	1732
	ssc-miR-146a-5p	13012	5145	2046	1704
	ssc-miR-30b-5p	11423	5569	4200	2780
	ssc-miR-31	9513	2276	3576	1154
	ssc-miR-215	3863	81	24	1268
	ssc-miR-30c-3p	2610	10670	6738	7063
	ssc-miR-2320-5p	2350	534	1128	462
	ssc-miR-345-3p	2181	742	974	776
	ssc-miR-664-5p	1553	613	741	447
	ssc-miR-205	911	379	305	179
	ssc-miR-129a	639	1331	307	89
	ssc-miR-4332	235	20	59	55
	ssc-miR-452	178	65	34	73
	ssc-miR-432-5p	161	48	20	27
	ssc-miR-628	128	62	30	11
	ssc-miR-301	97	24	26	25
	ssc-miR-382	37	7	9	11
