# Supplementary material for: Difference in microRNA expression and editing profile of lung tissues from different pig breeds related to immune responses to HP-PRRSV
Source: Sci Rep. 2015 Apr 9;5:9549. doi: 10.1038/srep09549 (PMC5381705; doi:10.1038/srep09549)
Supplement: Supplementary Information [file srep09549-s1.pdf]

Difference in microRNA expression and editing profile of lung tissues from different pig breeds related to immune responses to HP-PRRSV

Jia Li[1], Zhisheng Chen[1], Junlong Zhao[2], Liurong Fang[2], Rui Fang[2], Jiang Xiao[2], Xing Chen[2], Ao Zhou[2], Yingyin Zhang[2], Liming Ren[1], Xiaoxiang Hu[1], Yaofeng Zhao[1], Shujun Zhang[2]*, Ning Li[1]*

[1]State Key Laboratory for Agrobiotechnology, China Agricultural University, Beijing, 100193, People's Republic of China

[2]Key Laboratory of Agricultural Animal Genetics，Breeding and Reproduction of Ministry of Education, Huazhong Agriculture University, Wuhan, 430070，People's Republic of China

Corresponding author:

Ning Li

Tel: 008610-62733323

Fax: 008610-62733904

E-mail: ninglcau@cau.edu.cn

Co-corresponding author:

Shujun Zhang

Tel: 0086-27-87515340

Fax: 0086-27-87515340

Email: sjxiaozhang@mail.hzau.edu.cn

Supplementary Materials

Supplementary Figure S1 The length distribution of sequencing reads

Supplementary Figure S2 Small RNA annotation

Supplementary Table S1 Novel microRNAs predicted by mireap 2.0

Supplementary Table S2 Top 10 up-/down-regulated microRNAs at 3, 5, 7 dpi compared with control individuals (0 dpi) in Tongcheng and Landrace pigs' lungs

Supplementary Table S3 GO term (Biological Process) enrichment analysis of breed specific common DEmiRNAs' target genes (Top five)

Supplementary Table S4 KEGG enrichment analysis of breed specific common DEmiRNAs' targets

Supplementary Table S5 Accession number of the genome of the eight PRRSV strains

Supplementary Table S6 14 microRNAs predicted to bind to conserved regions of WUH3 genome

Supplementary Table S7 MicroRNAs editing level in the lungs of Tongcheng and Landrace pigs

Supplementary Table S8 MicroRNA editing sites number

Supplementary Table S9 Primers for qRT-PCR

Supplementary Table S10 The overlapped DEmiRNAs between the two breeds

A

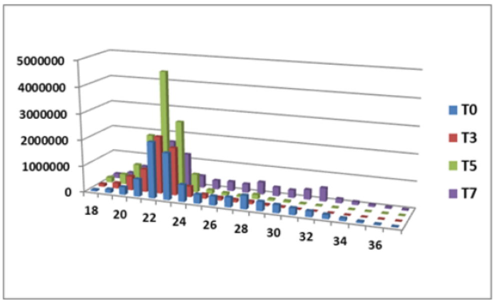

B

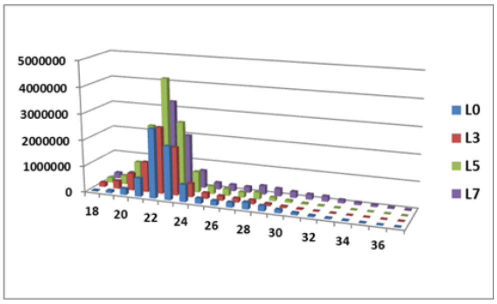

Supplementary Figure S1.The length distribution of sequencing reads

The x-axis represents the length (bp) of reads; The y-axis represents the number of reads. T0 : small RNA library of Tongcheng lung tissues at 0 dpi; T3 : small RNA library of Tongcheng lung tissues at 3 dpi; T5: small RNA library of Tongcheng lung tissues at 5 dpi; T7: small RNA library of Tongcheng lung tissues at 7 dpi; L0: small RNA library of Landrace lung tissues at 0 dpi; L3: small RNA library of Landrace lung tissues at 3 dpi; L5: small RNA library of Landrace lung tissues at 5 dpi; L7: small RNA library of Landrace lung tissues at 7 dpi;

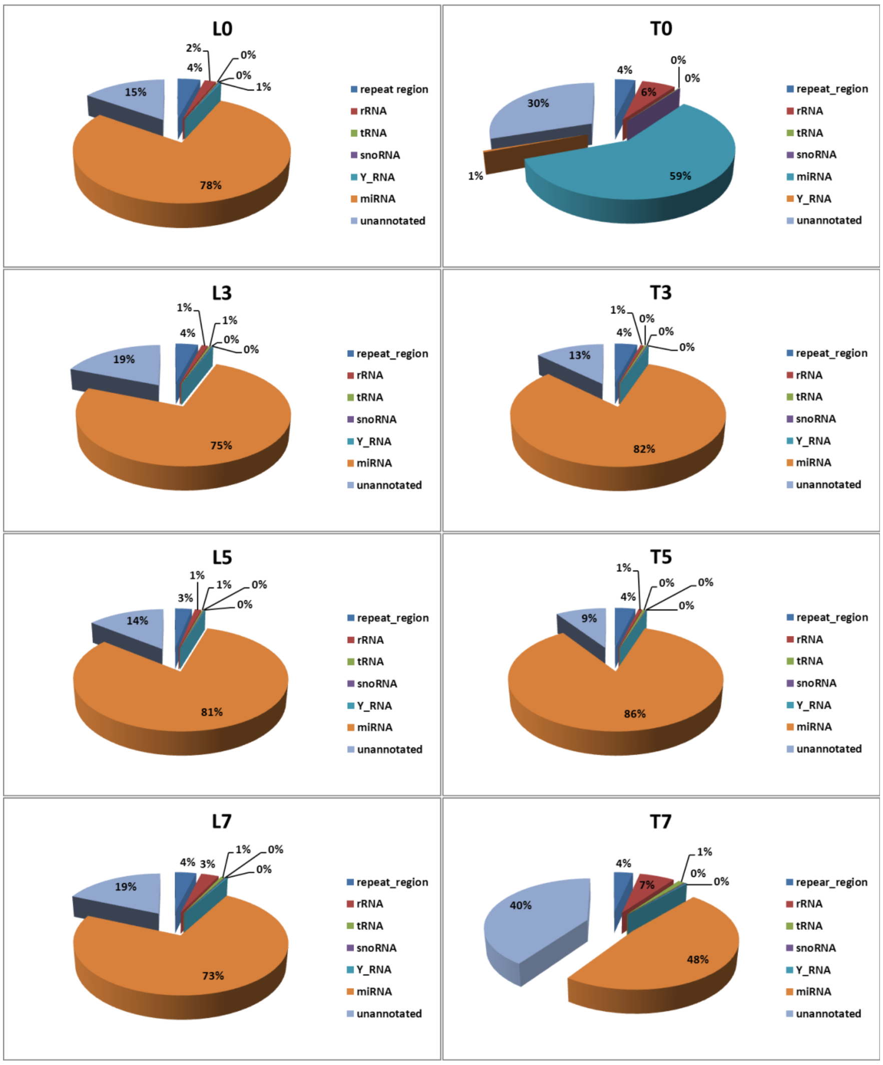

Supplementary Figure S2. Small RNA annotation

Supplementary Table S1. Novel microRNAs predicted by mireap 2.0

Chr	Start	End	Mature Sequence	Reads
1	17377819	17377841	TCCCTGTGGTCTAGGGGTTAGGA	118
1	24218214	24218236	ATGGACTAACTGTGGTATTGGGA	32
1	25302016	25302037	TTCAGGTAAAAGACACACACTA	14
1	28530870	28530893	ATAGCCCTTCTTAGTCCACATGTT	13
1	32473196	32473218	GACATATTGATTGAAGCAGTTCT	62
1	38099174	38099194	ACAGCAAAAACTACTACCTCT	16
1	56916489	56916509	AGGCCCTTACATAGTCAGACT	12
1	56916530	56916550	GTCTGACTATGTAGGGGCCTA	11
1	95108130	95108153	AACAGCTGTAGCAAAGGAAGTATG	81
1	108448184	108448206	TTGCTCAGAATAGTTTCCTTTCA	14
1	123239660	123239682	ATCAAGATTTGTAGGAAGAAGGA	15
1	154994061	154994083	AAACCTGCCCTCTCTGACAGTCC	33
1	156655882	156655903	TACCCCATGTGCAGCCTCTTCT	885
1	182229624	182229647	CGGAGTGGAATTTCATCATTGCTG	12
1	182942511	182942531	CACACCCCAGACCCAACGGCA	32
1	187427808	187427831	TCCCTGGTCTAGTGGTTAGGAATC	28
1	201953191	201953214	AGACCGGGGACCGGAGCGAGCAGA	97
1	217555245	217555265	TGTGACTGTGGCGTACGCTGG	54
1	227405749	227405772	TAACGGACAGATACGAGGCAGACA	131
1	231280703	231280723	ACTCAAGTAGAGGTTATGCTT	16
1	231280704	231280724	CTCAAGTAGAGGTTATGCTTT	15
1	261330938	261330961	TCCCTAGTGGTTAGGATTCAGCAC	25
1	261330939	261330960	CCCTAGTGGTTAGGATTCAGCA	451
1	278755796	278755816	CTGGTCCAGTCTACTCTTGTC	57
1	301364187	301364208	AGGGGTCTGTTCTCTCCCTGAA	53
1	306508991	306509013	AGAGTGACGGAGCTGAGGTGCGA	36
1	307112383	307112404	AATTTGGGGAAGGCTGTATTCA	95
2	7361076	7361097	TCGGACTCAGGACGGGCCTTGA	49
2	7444711	7444732	TCACTTCAGAGGAGCCAACAAA	133
2	8531520	8531541	GGGCAAGCCTGCGGAGGTGTGG	11
2	8587799	8587820	AAGTCCCGACTGTCGTCATCGT	16
2	9700083	9700104	AAACGATGATGGAAGGTGCCGA	106
2	28658564	28658586	TGTTCTTCTTGCAGCGGAAAAGA	12
2	47819332	47819354	TGCTGAGCTGCGTGGGCTTGCTG	60
2	49967036	49967056	TGGGGGACTGTGAACAGCGGG	12
2	65111800	65111822	AGCTAAGTTGGAAGAGTGCTATG	25
2	71114373	71114393	GATCCCTCTGCTATCCCCCAG	24
2	73210514	73210536	CTCTGCCCGCTCTCTGTCTTACA	34
2	75243378	75243401	AGCAGACATTCCATAGACTGTATT	30
2	79261285	79261308	GTGATGGTCGAGACAGTGGCGAGG	14
2	79261286	79261307	TGATGGTCGAGACAGTGGCGAG	64

2	82423680	82423698	CCCTGCAAGGCCAGTCCTC	21
2	83026823	83026846	TACACCCTCTTGCTCATTCAGTCT	13
2	88902992	88903013	CCCTGCGTGGCTTCTCTGTGCA	28
2	88902993	88903015	CCTGCGTGGCTTCTCTGTGCAGT	71
2	88902995	88903016	TGCGTGGCTTCTCTGTGCAGTC	96
2	88903034	88903055	CACAGGAAGGCCACGGAGGGGC	148
2	140157754	140157775	TCTCAGACAGAGGGGCTCCACA	26
3	2180666	2180685	ACCCAGGACCTTAGGGCGGG	26
3	10664433	10664453	GGGGGAACTCCCAGACCAGCT	31
3	10664434	10664455	GGGGAACTCCCAGACCAGCTTC	430
3	10664470	10664492	AGCTGGTCTGGGAGTTCCCGGGT	105
3	18547277	18547300	TCTCGTCCGAGTGCGACAGCTTGT	13
3	59037906	59037926	CCTGTGCTTCCTCACCTGCAG	12
3	61902078	61902098	AGGCTGCGGAGGAGTCATGGC	31
3	84315293	84315316	AGCAGTTGTGGTACAGATCTTCGG	18
3	110277809	110277828	AGGACCAACACTCTCATATC	20
3	134560437	134560458	CCTCGTAGCTTGCTCACCAAAT	169
4	1049675	1049698	GCGCCGCGCTGGGAGCCCTGCGGA	13
4	38941049	38941070	TATTATCTGTGGGATACTCGGT	284
4	52062177	52062196	GTCTACGGCTATACCACCCT	11
4	56174511	56174531	TTCCTGAGTCGGACTGGGCTG	335
4	82680413	82680434	TGGCGCTGAGGAGAGAATTGCT	30
4	86976573	86976592	AGAGGAGGAAAACAGAGCCA	14
4	87278077	87278098	ACAGCTGCCAACCTTGACACTG	17
4	91061802	91061823	ATTGTCTTCTTCCCTTTTCCTA	51
4	92674228	92674250	AACAGAGACTGAGAAGAGTAGTA	29
4	97729322	97729345	AGATTGTAGAATGGGAGTTTAGTG	11
4	102804745	102804768	AGACCTTGATGGCTGGCTGAGTCT	216
4	108993456	108993476	GTGAGTTTGTAGGGTTGGAGA	13
4	117890751	117890772	CAAGAAGGAAGCGTCCCGACTG	82
4	121166770	121166791	AGTGATGATGATATAACTGCCT	1051
4	131749102	131749123	TCTGGCTTAGTCTGCAACTACA	12
4	132441563	132441584	TCAGAAGTGGGTCCAGGAATCT	73
4	139660446	139660467	AGCTGGACTCAGAATTTGTGCA	361
4	139664274	139664297	AGCAAGAGAGGACGATGGCTCTTA	74
5	956867	956890	TTTGGGACGCAGCAGGCATTCTCG	67
5	1499762	1499783	ACGCCCTTCCCCCCCTTCTTCA	218
5	3528413	3528434	CGGGCTGCGTGTCCCTCGGCAG	90
5	5388521	5388541	ATGTCCGCGGGTTCCCTATCC	4100
5	5388562	5388582	ATGCGGAACCTGCGGATACGG	242666
5	9143679	9143700	CGGCAGCTGCTATCAGGTTCAA	51
5	16196976	16196995	ACTGTCACGTGGGACAGGTG	41
5	22636826	22636846	AGGCCAAACAGAATGAACGCA	350
5	22753423	22753444	CTGCGCTTACTTCCACTTCCGG	106
5	27807457	27807478	CCCAGAAGCAGGTCGTCTAACA	254
5	30185309	30185329	ATGCCCAGGTCACATCTCAGA	367

5	31460173	31460196	AGGACTGTAGATCCTTAGGTCTCT	531
5	37317373	37317396	TATATTCATTCCGTGCAGTTCTTT	18
5	47395182	47395204	AAAGGCAGAATTATACTGACAGA	240
5	56818511	56818534	TCCCTGGTCTAGTGGTTAGGAGTC	18
5	57948016	57948036	AAGGGCTGCCTGGGAAGATCG	11
5	57948930	57948951	TCCAGCTGTGGAAGGAGCCATG	80
5	61513847	61513866	AGAGACTGTCTCACTGGAGA	136
5	62371284	62371305	TCGGGGAGGCTGTGCAGCGCGG	51
5	65301871	65301893	ATGACGTGAGGTTCTGCTCCCGA	66
5	65301872	65301893	TGACGTGAGGTTCTGCTCCCGA	52
5	65810441	65810464	ATGGGGCTTTGCCAGAACAGATGA	112
5	69621003	69621025	TCTGGGACTGAAGGCCGCGGCCT	19
5	77832012	77832031	GAAACCAATCCTTTTCATTT	103
5	79348529	79348549	ACCAACGTGGATACCCCGGGA	55
5	79349749	79349769	TCCAACGTGGATACCCCGGGA	54
5	79410562	79410585	AACTGTCTTTTCCTGAAATGTTGC	11
5	82017216	82017237	CAGCTCCGAGAAGGCGGGATGA	11
5	92993603	92993624	AGTGTCTACTGTAAACACGTCT	12
5	92993636	92993657	AGACGTGTTTACAGCAGACAAG	13
5	94001383	94001403	AAAAGCTGCTTGAGAGGGAGA	32
5	109259471	109259493	CCATTCCTTCGTCTGTGCACTAG	390
5	109259509	109259531	TAGGGCACAGGATGGGATGAGGA	813
5	109289733	109289755	ATGTGCATGTAGATGAAGACAGG	24
5	111999768	111999791	AACATGGCTAGAAAAGAGAATGAA	15
5	148078068	148078090	TGTGACTGTGGCGTACGCCGGCA	188
6	956867	956890	TTTGGGACGCAGCAGGCATTCTCG	67
6	3528413	3528434	CGGGCTGCGTGTCCCTCGGCAG	90
6	27807457	27807478	CCCAGAAGCAGGTCGTCTAACA	254
6	56818511	56818534	TCCCTGGTCTAGTGGTTAGGAGTC	18
6	57948016	57948036	AAGGGCTGCCTGGGAAGATCG	11
6	57948930	57948951	TCCAGCTGTGGAAGGAGCCATG	80
6	62371284	62371305	TCGGGGAGGCTGTGCAGCGCGG	51
6	65301871	65301893	ATGACGTGAGGTTCTGCTCCCGA	118
6	79348529	79348549	ACCAACGTGGATACCCCGGGA	55
6	79349749	79349769	TCCAACGTGGATACCCCGGGA	54
6	79410562	79410585	AACTGTCTTTTCCTGAAATGTTGC	11
6	82017216	82017237	CAGCTCCGAGAAGGCGGGATGA	11
6	94001383	94001403	AAAAGCTGCTTGAGAGGGAGA	32
6	111999768	111999791	AACATGGCTAGAAAAGAGAATGAA	203
7	1383027	1383049	TCGGGTGAAAGCGGTCAGGTTCA	28
7	1581831	1581852	AACAAGGTCATTGGGGAGTTTC	36
7	4037112	4037132	AGGCGGGTGCTCGATGCTAGA	26
7	4037114	4037134	CATCTAGCATCGAGCACCCGC	48
7	4428747	4428768	GCACCAGACTTGCCCTCCCACG	138
7	4428748	4428767	CACCAGACTTGCCCTCCCAC	31
7	8701439	8701460	TCTGGTCCAGACACTGTGGAGC	14

7	20400309	20400331	TGGGGTGTGCTCAGAGCAGGAGG	11
7	20676043	20676066	TGCTATGTATTGTCAGTGCCTGTT	74
7	20917686	20917705	ACTTACTCTCAGAGTCGTGC	30
7	22962952	22962973	TAGCCAGTTTGGGAAGAATGCT	19
7	22962994	22963016	TGGATTGTTCTCCAACCTGGCTC	50
7	24367080	24367101	AGCAGGGGGTGTAGCTCAGTGG	525
7	27236502	27236523	CTGCGGACTGTGAGAATGCGGA	24
7	36178794	36178815	AGGCCTGTCCAATCCATCCTTT	25
7	37088414	37088436	TGTCATGCTGGGGAGTGTAGTGA	93
7	41758989	41759012	CACCCTTTCCTGTGCCCTTTTAAG	11
7	43704066	43704087	ATGAGATGCAGAGCTTGGATGA	76
7	55687420	55687442	TAAGAGCATCTGAACCAGGGGTT	129
7	57212966	57212989	TCTTTCCCCGCTGATTCCACCAAG	361
7	58609073	58609092	TGCATGGTCTTGGTCTCGCA	16
7	58774361	58774380	CACCTCTTCCAACACTGACT	64
7	69400041	69400062	GGCACCATTCTACTTTCTGTCT	83
7	71609590	71609613	AGCTGAAGTGTTGGAAAGATATAA	22
7	83112645	83112666	CCTCGATGATGAGTTGCCATGC	20
7	93541810	93541831	TCCCTGGTGGTTAGGATTCAGC	37
7	106126682	106126702	ATTCTGGATGGACTGTAAGTA	26
7	116871333	116871355	GAAATATGATGTACAGCCAGGTT	20
7	120648791	120648811	TGGTCAAGGCCTGTCGCCGCT	60
7	122371914	122371935	CCAGAGGTTTCTAGTTCACAGA	84
7	122371916	122371935	AGAGGTTTCTAGTTCACAGA	83
7	128633075	128633097	AGAGGAATCTCTGGGCGCTGAAG	11
7	128881150	128881173	GATCTGATAAATGCACGCACCCCC	65
7	131276640	131276661	TCGGACTCAGGACGGGCCTCTA	16
7	147811959	147811981	AGCATACAGGTCTGACTATAAGG	15
8	1383027	1383049	TCGGGTGAAAGCGGTCAGGTTCA	28
8	1581831	1581852	AACAAGGTCATTGGGGAGTTTC	36
8	41758989	41759012	CACCCTTTCCTGTGCCCTTTTAAG	11
8	57212966	57212989	TCTTTCCCCGCTGATTCCACCAAG	361
8	58774361	58774380	CACCTCTTCCAACACTGACT	64
8	71609590	71609613	AGCTGAAGTGTTGGAAAGATATAA	22
8	106126682	106126702	ATTCTGGATGGACTGTAAGTA	26
8	122371914	122371935	CCAGAGGTTTCTAGTTCACAGA	167
8	147811959	147811981	AGCATACAGGTCTGACTATAAGG	15
9	3719786	3719808	ATCTGATCCTGAAGAGCAGCTTA	21
9	7558767	7558789	TCTCCCACCCTTGCTGCCTGCAG	11
9	44168468	44168488	TGCACCCTTGCGTCGCCCTTG	29
9	100449453	100449476	TGCTCATCTTCGTGTAACCAGTCT	36
9	127780954	127780976	TGCTCATCACTGTCAAGTCTGCT	16
10	31964005	31964026	TCTTCGCTCCCTACAACCTCGT	28
10	54171491	54171513	AGCGAGGAACTGAGGAAGGTATG	21
11	4056393	4056413	CTGCACAACAGAGTGAGTCAG	108
11	14607183	14607204	TCTGCTGGAGGACGCCCGGTGT	17

11	14607184	14607204	CTGCTGGAGGACGCCCGGTGT	75
11	25423189	25423210	TCCGTTTCTCTTCCGCGTGCAA	219
11	75694933	75694954	CGCTGTCTTGAGAACTTTGCCA	35
11	81643736	81643758	ACTTTCCCGGGATTTGGAGCGCT	89
11	84943665	84943685	TCCTGGAGGACGTGCTGTGCC	21
12	7922011	7922033	CCTCCCTGGGAGCATCTCTGGGT	18
12	14702253	14702275	AGAGGAGCAGCGTGGACCCACCT	29
12	18750388	18750408	CTACTCTGTCCCCACCTCCAG	112
12	20165413	20165432	CTCTGCCTTTACAATCCCGT	11
12	20179161	20179182	TCGCCGTTCCATTGCTCCCAGT	77
12	20320700	20320722	TGACATCTCTGTGAGTTCTCGAT	12
12	26716564	26716584	TCTGCTTGAGCAGGAGCCTCT	30
12	34136284	34136306	TGCAGGAAAAATAGCCCCTCGTG	20
12	34137075	34137096	AATCCTCTGGTCTGGCTGGAGA	162
12	38333515	38333537	ACAGCGCAATCCCCTCGTCCTGT	57
12	38333517	38333537	AGCGCAATCCCCTCGTCCTGT	603
12	39743423	39743444	AAGCTGGACGTCCTGTGCCGCT	13
12	44808953	44808975	CCTGGTCTAGTGGTTAGGATTTG	27
12	45717317	45717337	TCCCTGTGGTTTAGTGGTTAG	123
12	45971995	45972014	CCGATGGACGGTCCCCACAG	20
12	46942594	46942614	TGCATATGATGGAAAAACTTG	390
12	46942595	46942614	GCATATGATGGAAAAACTTG	72
12	55878371	55878390	TTACAGTATTAGTCGCTTTT	92
12	55975082	55975103	TCAAGAGACGGATCCTGGAAGT	21
13	20801320	20801341	ATCGGAAAATAGGAGATGTGCC	53
13	24885297	24885316	CCTATCCTGGGTTACTTGAA	5644
13	24996849	24996871	CAGCGCATGCTCGTCCTAACCCA	24
13	26080904	26080924	TGGGCAATGCACAGCTCCAAT	79
13	26080943	26080964	CTTGGAATTTTGCAGTGTCCAC	77
13	26080944	26080965	TTGGAATTTTGCAGTGTCCACC	161
13	27666079	27666101	TGGGCTGCCAGATCTGGGGCATG	25
13	33474519	33474541	TTTGCTCTGCTCCTGCCACATGC	106
13	36038252	36038271	CCCCATCAGTCTGCTAAGCT	31
13	74637346	74637367	TCCAGTGTTGGGAACGCGGACG	15
13	75927582	75927604	GAGCTGTAGGTCGGGTGTCGTCT	24
13	80688705	80688726	TGTATCTGCGACCTAGACCCTA	109
13	108096475	108096495	TCAGTAGTTGCATGCAGGGAG	11
13	108992619	108992642	CACCTGTGATGTGAGATGTAAATC	63
13	117009658	117009678	AAGTGTCTGAGCCGAGTCCCA	23
13	130797293	130797316	AAGCAGGATATAGTTACAATGTAG	28
13	131653413	131653434	TTCCACTGATGACTTCTTGTTA	26
13	149704339	149704358	TCAGACCAGACTGCCCAGCT	24
13	172694568	172694591	CATAGCCTAGATCTCTTATTTGCT	32
13	190079543	190079564	TCTTGCATTTTTCTAACAGAAT	45
13	217449316	217449339	ATGTTGTCACACTCGCAGAACTGG	26
14	42019585	42019606	GAGCATGATGGCAATTCTGAGG	21

14	60676765	60676785	TGCGGGTGAGACGGAAGAACA	34
14	66245955	66245976	TTGGAGCTATGGTGAAAGGCAT	11
14	88206369	88206389	CTGGGTTGAATGGCTGGTTCT	96
14	88206370	88206389	TGGGTTGAATGGCTGGTTCT	47
14	88819648	88819668	AATGGGCTCTTAGAAACTGGT	12
14	98535259	98535281	TTCCAGCAGTAGTCAGCTGTCAG	161
14	118986507	118986529	AGCATTTTGAACTTGAGGACAGA	13
14	121669993	121670013	GTGAGTAGGACGGAGGTCAGG	52
14	125224385	125224406	TCGAGATGCCCAGCCTTCCAGT	15
14	133619339	133619360	ACAGGCACGGCTGGTTTGAGCA	31
14	133619375	133619394	CCAAACCAGTTGTGCCTGTA	47
14	134551201	134551222	TCAGGTATAGCTGGATCCAGGG	12
14	135881799	135881819	CATCTTCCTCCCACTGTCCTT	49
14	152293999	152294020	TGGACGGCTGGATGGACAGTCA	14
15	87076860	87076879	ATCTGAGACTGGGGAAGGTT	15
15	122437639	122437662	AGCATAATCCTCGCTGTCCTTGAG	30
15	133359011	133359032	GAGCATGGTAATGGATTTATGG	162
15	133415452	133415473	GAGCATGGTAATGGATTTATGG	162
15	151429245	151429267	ACGGTTTGGACGTTAAGAAGGAT	59
16	18362938	18362958	TTGGTGTACACTGGAATAGCT	41
16	51742263	51742282	AAGCCTACAGCACCCGGTAT	1262
16	51894829	51894850	CGCGTTGGTGGTATAGTGGTGA	67
16	55771001	55771022	AAGACCGTCTGCTGAACTCAGC	16
16	59135350	59135372	GCTTGACTGTGGACATGTGCATC	12
16	59466905	59466926	TCTGCCTGTAGGAATGCTGTAG	59
16	75076928	75076950	AATCCAGGCAGTAGAGCAGGTGT	140
17	5343465	5343487	GCGGTGAAGGTAGGACTACCGGT	17
17	5625189	5625208	CTCCCTCGGCCCCGGGATCC	25
17	5670601	5670620	CTCCCTCGGCCCCGGGATCC	25
17	42437612	42437634	TGTGACTGTGGCGTACGCTGGCA	188
17	65136727	65136747	ACGCGTGAGCCACCTCGCCTC	35
17	66288750	66288770	ACAGGATTGGGGGGGGCCCTC	55
18	1867513	1867532	GCTCCCGCCGGCTTCTCCTG	178
18	6263311	6263333	AAAGGCAGTCCGGGCTGGACATT	11
18	10165789	10165812	CTCTGGTTTGACTTAGCATATTCA	221
18	37221469	37221489	GAAATGATGAACTGTCTTAGG	44
18	45517586	45517609	AGCTGAGATTGTAGCATGGGTATA	15
18	55400247	55400267	CTGCAAGCCTCCAGCGTGCTT	16
GL892805.1	128571	128593	TTGAACCCAGTGAAACCAGGTCT	49
GL892848.2	60391	60411	TGGTCAAGGCCTGTCGCCGCT	60
GL894032.2	21866	21885	GCATATGATGGAAAAACTTG	72
GL894109.2	9419	9440	ACGCCCTTCCCCCCCTTCTTCA	218
GL894109.2	38822	38844	TCCTGGTGGTGCCCTTCCTGCAA	1025
GL894257.1	12190	12209	GCCCTAGGCCATGCTCCCCA	21
GL894542.2	9144	9166	TTTGCTCTGCTCCTGCCACATGC	86
GL894542.2	9146	9166	TTTGCTCTGCTCCTGCCACAT	20

GL895967.2	49153	49174	CCAGCTGTGGAAGGAGCCATGC	70
GL895967.2	49154	49175	TCCAGCTGTGGAAGGAGCCATG	18
GL896221.1	9916	9938	AGATACACAGACTTTTCTTCAAG	14
GL896264.1	37171	37192	ATCTCAGGTGTGTCAGCCCGAG	13
GL896264.1	37172	37192	ATCTCAGGTGTGTCAGCCCGA	41
GL896425.1	1752	1771	GGTGCCTGACGTCTTGGCAG	16
GL896441.1	1135	1156	TAGCTGCATGGCTGGTGCGCCA	12
JH118494.1	36737	36757	ATGCCCAGGTCACATCTCAGA	367
JH118585.1	53415	53436	TCTGGTCCAGACACTGTGGAGC	14
JH118729.1	80985	81006	CCCTGCGTGGCTTCTCTGTGCA	28
JH118729.1	80986	81008	CCTGCGTGGCTTCTCTGTGCAGT	71
JH118729.1	80988	81009	TGCGTGGCTTCTCTGTGCAGTC	96
JH118729.1	81027	81048	CACAGGAAGGCCACGGAGGGGC	148
JH118788.1	3167	3188	AGGCCTGTCCAATCCATCCTTT	25
X	41173119	41173142	AGTAGTTTGGGTGGAAGAGTCAGA	12
X	41391987	41392007	AGGCCTGTCCAACAGATGCAT	30
X	51475500	51475522	CCTTCCTCAGCCCACTTCCTCCA	11
X	53138319	53138341	CACAACTCTGGGAAGTACCATTT	2065
X	53138359	53138380	AATGGTGCTCCAGGGTTGTACA	105
X	126559235	126559257	AATGCCTACCTTCCTTTTGGATT	13
X	141842739	141842761	CAGAGGCCAGAGGGCAGGACGCT	19
X	143364497	143364516	TTGGACAGCCCGGAGGACCT	28

Supplementary Table S2. Top 10 up-/down-regulated microRNAs at 3, 5, 7 dpi compared with control groups in Tongcheng and Landrace pigs' lungs

**Landrace**	**MicroRNA**	**0 dpi (TMM)**	**3 dpi (TMM)**	**Log2FC**	**FDR**	**Tongcheng**	**0 dpi (TMM)**	**3 dpi (TMM)**	**Log2FC**	**FDR**
**Up**	ssc-miR-183	65	1961	4.93	4.74E-16	ssc-miR-490-5p	6	27	2.15	5.35E-04
	ssc-miR-342	11	70	2.76	2.33E-11	ssc-miR-1343	12	49	2.03	3.54E-06
	ssc-miR-30c-3p	2610	10670	2.03	4.74E-16	ssc-miR-574	42	161	1.95	5.25E-16
	ssc-miR-504	50	200	2.02	4.74E-16	ssc-miR-342	21	68	1.73	1.10E-06
	ssc-miR-193a-5p	281	1009	1.85	4.74E-16	ssc-miR-183	87	270	1.63	5.25E-16
	ssc-miR-193a-3p	919	3156	1.78	4.74E-16	ssc-miR-219	31	91	1.55	9.51E-08
	ssc-miR-331-3p	1084	3657	1.75	4.74E-16	ssc-miR-30c-3p	3559	10240	1.52	5.25E-16
	ssc-miR-143-3p	192555	578719	1.59	4.74E-16	ssc-miR-331-3p	1344	3773	1.49	5.25E-16
	ssc-miR-221-5p	1672	4421	1.40	4.74E-16	ssc-miR-424-5p	1910	5315	1.48	5.25E-16
	ssc-miR-28-3p	541	1274	1.24	4.74E-16	ssc-miR-143-3p	119229	322185	1.43	5.25E-16
**Down**	ssc-miR-215	3863	81	-5.58	4.74E-16	ssc-miR-4332	316	18	-4.19	5.25E-16
	ssc-miR-218-3p	29	2	-4.53	4.39E-07	ssc-miR-144	92	13	-2.91	1.05E-15
	ssc-miR-4331	19	2	-3.89	1.88E-04	ssc-miR-382	21	4	-2.73	8.58E-04

	ssc-miR-4332	235	20	-3.58	4.74E-16	ssc-miR-1839-3p	56	12	-2.32	7.25E-08
	ssc-miR-19b	97	15	-2.71	8.76E-16	ssc-miR-95	681	140	-2.29	5.25E-16
	ssc-miR-382	37	7	-2.58	5.41E-06	ssc-miR-500	306	65	-2.26	5.25E-16
	ssc-miR-144	193	34	-2.54	4.74E-16	ssc-miR-2320-5p	1200	261	-2.20	5.25E-16
	ssc-let-7i	580833	105860	-2.46	4.74E-16	ssc-miR-664-5p	1299	305	-2.09	5.25E-16
	ssc-miR-15a	3476	643	-2.44	4.74E-16	ssc-miR-155-5p	866	228	-1.93	5.25E-16
	ssc-miR-545-3p	33	7	-2.39	4.08E-05	ssc-miR-374b-3p	864	242	-1.84	5.25E-16
**Landrace**	**MicroRNA**	**0 dpi (TMM)**	**5 dpi (TMM)**	**Log2FC**	**FDR**	**Tongcheng**	**0 dpi (TMM)**	**5 dpi (TMM)**	**Log2FC**	**FDR**
**Up**	ssc-miR-183	65	2588	5.33	4.33E-16	ssc-miR-183	87	605	2.80	4.97E-16
	ssc-miR-30c-5p	111	3615	5.03	4.33E-16	ssc-miR-34a	153	813	2.41	4.97E-16
	ssc-miR-532-5p	8655	59685	2.79	4.33E-16	ssc-miR-490-5p	6	30	2.28	6.81E-05
	ssc-miR-143-3p	192555	1146757	2.57	4.33E-16	ssc-miR-193a-3p	1139	5124	2.17	4.97E-16
	ssc-miR-193a-3p	919	4606	2.33	4.33E-16	ssc-miR-143-3p	119229	460651	1.95	4.97E-16
	ssc-miR-221-5p	1672	6813	2.03	4.33E-16	ssc-miR-424-5p	1910	7141	1.90	4.97E-16
	ssc-miR-148a-5p	56	174	1.66	5.49E-15	ssc-miR-345-5p	52	191	1.89	4.97E-16
	ssc-miR-219	40	112	1.52	7.33E-09	ssc-miR-551a	108	357	1.73	4.97E-16
	ssc-miR-210	1517	4090	1.43	4.33E-16	ssc-miR-219	31	97	1.64	5.27E-09
	ssc-miR-34c	439325	1142294	1.38	4.33E-16	ssc-miR-1306-3p	384	1144	1.58	4.97E-16
**Down**	ssc-miR-215	3863	24	-7.36	4.33E-16	ssc-miR-374b-3p	864	13	-6.07	4.97E-16
	ssc-miR-122	55623	5801	-3.26	4.33E-16	ssc-miR-1839-3p	56	2	-5.76	2.61E-14
	ssc-miR-432-5p	161	20	-3.02	4.33E-16	ssc-miR-4332	316	12	-4.81	4.97E-16
	ssc-miR-1839-3p	65	9	-2.87	1.35E-11	ssc-miR-142-5p	12035	966	-3.64	4.97E-16
	ssc-miR-95	518	72	-2.86	4.33E-16	ssc-miR-450b-5p	49	8	-2.70	5.06E-08
	ssc-miR-146b	13667	2078	-2.72	4.33E-16	ssc-miR-145-3p	322	52	-2.65	4.97E-16
	ssc-miR-146a-5p	13012	2046	-2.67	4.33E-16	ssc-miR-423-5p	32751	6137	-2.42	4.97E-16
	ssc-miR-126-5p	13575	2546	-2.41	4.33E-16	ssc-let-7d-3p	248	47	-2.40	4.97E-16
	ssc-miR-452	178	34	-2.40	4.33E-16	ssc-miR-1306-5p	189	38	-2.32	4.97E-16
	ssc-let-7i	580833	115555	-2.33	4.33E-16	ssc-miR-374a-3p	3076	794	-1.95	4.97E-16
**Landrace**	**MicroRNA**	**0 dpi (TMM)**	**7 dpi (TMM)**	**Log2FC**	**FDR**	**Tongcheng**	**0 dpi (TMM)**	**7 dpi (TMM)**	**Log2FC**	**FDR**
**Up**	ssc-miR-183	65	412	2.68	3.87E-16	ssc-miR-215	20	1400	6.20	4.30E-16
	ssc-miR-143-3p	192555	974013	2.34	3.87E-16	ssc-miR-1307	135	1428	3.41	4.30E-16
	ssc-miR-101	2252	10815	2.26	3.87E-16	ssc-miR-19b	36	307	3.10	4.30E-16
	ssc-miR-374b-3p	54	258	2.26	3.87E-16	ssc-miR-182	77	436	2.52	4.30E-16
	ssc-miR-193a-5p	281	1211	2.11	3.87E-16	ssc-miR-143-3p	119229	626459	2.39	4.30E-16

	ssc-miR-148a-5p	56	218	1.98	3.87E-16	ssc-miR-210	893	4426	2.31	4.30E-16
	ssc-miR-4331	19	66	1.84	2.90E-07	ssc-miR-193a-5p	435	2091	2.27	4.30E-16
	ssc-miR-219	40	131	1.74	1.82E-12	ssc-miR-744	902	4018	2.16	4.30E-16
	ssc-miR-1307	229	758	1.73	3.87E-16	ssc-miR-183	87	364	2.07	4.30E-16
	ssc-miR-210	1517	4894	1.69	3.87E-16	ssc-miR-148a-5p	59	226	1.95	4.30E-16
**Down**	ssc-miR-122	55623	1063	-5.71	3.87E-16	ssc-miR-95	681	88	-2.96	4.30E-16
	ssc-miR-411	23	1	-4.58	4.32E-06	ssc-miR-129a	662	88	-2.92	4.30E-16
	ssc-miR-218-3p	29	2	-3.90	6.83E-07	ssc-miR-664-3p	424	59	-2.86	4.30E-16
	ssc-miR-628	128	11	-3.59	3.87E-16	ssc-miR-1839-3p	56	10	-2.60	7.00E-09
	ssc-miR-1839-3p	65	6	-3.47	2.29E-13	ssc-miR-374a-3p	3076	527	-2.55	4.30E-16
	ssc-miR-195	26235	2585	-3.34	3.87E-16	ssc-miR-628	79	14	-2.50	3.95E-12
	ssc-miR-150	1997	215	-3.22	3.87E-16	ssc-miR-1306-5p	189	36	-2.41	4.30E-16
	ssc-miR-664-3p	280	31	-3.18	3.87E-16	ssc-miR-125a	8213	1620	-2.34	4.30E-16
	ssc-miR-142-5p	8382	932	-3.17	3.87E-16	ssc-miR-374a-5p	4881	1026	-2.25	4.30E-16
	ssc-miR-15a	3476	394	-3.14	3.87E-16	ssc-miR-374b-5p	4159	881	-2.24	4.30E-16

Supplementary Table S3. GO term (Biological Process) enrichment analysis of breed specific common DEmiRNAs' target genes (Top five)

Tongcheng specific common DEmiRNAs GO enrichment (BP)

GO ID	GO Term	FDR
GO:0006355	regulation of transcription, DNA dependent	0.018548
GO:0030217	T cell differentiation	0.018548
GO:0035019	somatic stem cell maintenance	0.027894
GO:0010564	regulation of cell cycle process	0.027894
GO:0042769	DNA damage response, detection of DNA damage	0.018548

Landrace specific common DEmiRNAs GO enrichment (BP)

GO ID	GO Term	FDR
GO:0061383	trabecula morphogenesis	0
GO:0007186	G protein coupled receptor signaling pathway	9.94E-26
GO:0050896	response to stimulus	9.95E-26
GO:0051726	regulation of cell cycle	4.19E-07
GO:0007165	signal transduction	1.89E-06

Supplementary Table S4. KEGG enrichment analysis of breed specific common DEmiRNAs' target genes

KEGG enrichment of Landrace specific DEmiRNAs' target genes

	KEGGID	Pvalue	OddsRatio	ExpCount	Count	Size	Term	Symbols
1	5200	1.52E-11	3.961002719	14.24299	43	240	Pathways in cancer	MAPK9;TRAF6;STAT1;TGFBR1;HIF1A;KIT;FAS;NOS2;IL8;ITGB1;EGFR;SMAD4;VEGFA;TP53;ITGAV;IGF1R;FGF2;PPARG;FADD;MET;NFKB1;CDC42;XIAP;CHUK;RAD51;FOS;CDK4;RB1;CDKN1A;EGLN3;FZD3;E2F1;E2F3;PTEN;CRK;MLH1;CCNE2;FZD7;ERBB2;FZD4;CCNE1;FGF10;WNT1
2	5212	1.63E-08	7.665786315	3.026636	16	51	Pancreatic cancer	MAPK9;STAT1;TGFBR1;EGFR;SMAD4;VEGFA;TP53;NFKB1;CDC42;CHUK;RAD51;CDK4;RB1;E2F1;E2F3;ERBB2
3	4110	7.44E-08	5.128205128	5.103738	20	86	Cell cycle	SMAD4;TP53;CDC20;CCND3;CDK4;RB1;CDKN1A;E2F1;E2F3;MCM3;CDK1;TTK;E2F5;CCNA2;CHEK1;CCNE2;WEE1;CCNE1;CDC14A;CDKN2D
4	5219	3.34E-06	8.64322692	1.721028	10	29	Bladder cancer	IL8;EGFR;VEGFA;TP53;CDK4;RB1;CDKN1A;E2F1;E2F3;ERBB2
5	5222	5.61E-06	4.796427422	3.976168	15	67	Small cell lung cancer	TRAF6;NOS2;ITGB1;TP53;ITGAV;NFKB1;XIAP;CHUK;CDK4;RB1;E2F1;E2F3;PTEN;CCNE2;CCNE1
6	4115	1.03E-05	5.242890396	3.204673	13	54	p53 signaling pathway	FAS;TP53;CCNG1;CCND3;CDK4;CDKN1A;CDK1;PTEN;CHEK1;CCNE2;MDM4;PERP;CCNE1
7	5218	3.60E-05	4.941322314	3.085981	12	52	Melanoma	EGFR;TP53;IGF1R;FGF2;MET;CDK4;RB1;CDKN1A;E2F1;E2F3;PTEN;FGF10
8	5142	3.86E-05	3.744821872	5.163084	16	87	Chagas disease (American trypanosomiasis)	IRAK1;MAPK9;TRAF6;TGFBR1;FAS;NOS2;IL8;PPP2R2A;IL1B;CCL2;TLR4;FADD;NFKB1;IRAK4;CHUK;FOS
9	4620	4.59E-05	3.885329498	4.688318	15	79	Toll-like receptor signaling pathway	IRAK1;MAPK9;TRAF6;STAT1;IL8;IL1B;IFNA1;TLR4;FADD;NFKB1;IRF7;IRAK4;CHUK;FOS;TICAM2
10	5215	5.33E-05	4.061842105	4.213551	14	71	Prostate cancer	EGFR;TP53;IGF1R;CREB1;NFKB1;CHUK;RB1;CDKN1A;E2F1;E2F3;PTEN;CCNE2;ERBB2;CCNE1
11	5220	5.38E-05	4.703659976	3.204673	12	54	Chronic myeloid leukemia	TGFBR1;SMAD4;TP53;NFKB1;CHUK;CDK4;RB1;CDKN1A;E2F1;E2F3;CRK;GAB2
12	4621	6.23E-05	5.648671566	2.314486	10	39	NOD-like receptor signaling pathway	MAPK9;TRAF6;IL8;IL1B;CCL2;NFKB1;XIAP;NOD2;CHUK;TAB3
13	4520	8.20E-05	4.880324769	2.848598	11	48	Adherens junction	TGFBR1;PTPN1;EGFR;SMAD4;IGF1R;MET;CDC42;SRC;WASF1;NLK;ERBB2
14	4060	0.000263853	2.556393678	9.851402	22	166	Cytokine-cytokine receptor interaction	CXCR4;TGFBR1;CCL4;IL13;KIT;FAS;IL8;EGFR;IL1B;CD40LG;IL7;CCL2;IL15;IFNA1;CCR9;CXCL12;MET;IL6ST;TNFSF13B;TNFSF12;IL17RB;CXCL13
15	860	0.000344985	6.683257919	1.424299	7	24	Porphyrin and chlorophyll metabolism	HMBS;HMOX1;UROS;PPOX;FECH;CPOX;UROD
16	5140	0.000566721	3.75154321	3.501402	11	59	Leishmaniasis	IRAK1;TRAF6;STAT1;NOS2;ITGB2;ITGB1;IL1B;TLR4;NFKB1;IRAK4;FOS
17	5160	0.000660258	2.812086537	6.528037	16	110	Hepatitis C	MAPK9;TRAF6;STAT1;IL8;EGFR;PPP2R2A;TP53;IFNA1;NFKB1;IRF7;RNASEL;CHUK;CDKN1A;IFIT1;CLDN18;EIF2AK3
18	5214	0.00075105	4.313085234	2.551869	9	43	Glioma	EGFR;TP53;IGF1R;CDK4;RB1;CDKN1A;E2F1;E2F3;PTEN
19	4622	0.001425368	3.545972915	3.323364	10	56	RIG-I-like receptor signaling pathway	MAPK9;TRAF6;IL8;IFNA1;FADD;NFKB1;IRF7;CHUK;ISG15;DDX3X
20	4310	0.002073542	2.67248062	5.934579	14	100	Wnt signaling pathway	MAPK9;SMAD4;TP53;NFATC1;CCND3;FZD3;PRICKLE1;DKK1;NLK;FZD7;NFAT5;FZD4;TBL1XR1;WNT1
21	4810	0.003005673	2.25	8.901869	18	150	Regulation of actin cytoskeleton	ITGB2;ITGB1;EGFR;ITGAV;PPP1CB;SLC9A1;FGF2;RDX;MSN;CFL2;CDC42;WASF1;ITGA5;IQGAP1;CRK;PIP4K2A;FGF10;PAK1
22	4510	0.003191827	2.29535865	8.249065	17	139	Focal adhesion	MAPK9;ITGB1;EGFR;VEGFA;ITGAV;IGF1R;PPP1CB;MET;CCND3;CDC42;XIAP;SRC;ITGA5;PTEN;CRK;ERBB2;PAK1
23	4010	0.003583661	2.069742489	11.21636	21	189	MAPK signaling pathway	MAPK9;TRAF6;TGFBR1;FAS;EGFR;IL1B;TP53;FGF2;NFKB1;CDC42;CHUK;FOS;SIX4;SRF;CRK;NLK;MAP3K3;MAP3K12;STK3;FGF10;PAK1
24	5144	0.004359138	3.506042628	2.670561	8	45	Malaria	IL8;ITGB2;IL1B;CD40LG;CCL2;SELE;TLR4;MET
25	4722	0.00635244	2.509853783	5.341121	12	90	Neurotrophin signaling pathway	IRAK1;MAPK9;TRAF6;TP53;NFKB1;CDC42;IRAK4;SH2B3;PRDM4;CRK;MAP3K3;CAMK4

26	5223	0.007139946	3.537196356	2.314486	7	39	Non-small cell lung cancer	EGFR;TP53;CDK4;RB1;E2F1;E2F3;ERBB2
27	4380	0.007572812	2.445867769	5.459813	12	92	Osteoclast differentiation	MAPK9;TRAF6;STAT1;TGFBR1;IL1B;CREB1;PPARG;NFKB1;CHUK;FOS;GAB2;CAMK4
28	4210	0.007636075	2.709016393	4.154206	10	70	Apoptosis	IRAK1;FAS;PRKAR1A;IL1B;TP53;FADD;NFKB1;XIAP;IRAK4;CHUK
29	4360	0.010084996	2.451265573	4.985047	11	84	Axon guidance	CXCR4;ITGB1;NFATC1;CFL2;MET;CDC42;EFNB2;SEMA4D;NFAT5;L1CAM;PAK1
30	5145	0.010561692	2.3270366	5.697196	12	96	Toxoplasmosis	IRAK1;MAPK9;TRAF6;STAT1;NOS2;ITGB1;CD40LG;TLR4;NFKB1;XIAP;IRAK4;CHUK
31	5323	0.012301476	2.497477932	4.450935	10	75	Rheumatoid arthritis	IL8;ITGB2;IL1B;VEGFA;CCL2;IL15;TLR4;CXCL12;TNFSF13B;FOS
32	4012	0.012335069	2.652244898	3.798131	9	64	ErbB signaling pathway	MAPK9;EGFR;HBEGF;RPS6KB1;CDKN1A;SRC;CRK;ERBB2;PAK1
33	3030	0.014114137	4.022088353	1.483645	5	25	DNA replication	MCM3;PRIM1;RFC4;POLA2;FEN1
34	5100	0.015565099	2.974216919	2.670561	7	45	Bacterial invasion of epithelial cells	ITGB1;MET;CDC42;SRC;WASF1;ITGA5;CRK
35	4666	0.018094503	2.639455782	3.38271	8	57	Fc gamma R-mediated phagocytosis	CFL2;CDC42;RPS6KB1;WASF1;CRK;GAB2;MARCKS;PAK1
36	5211	0.019510174	2.824089069	2.789252	7	47	Renal cell carcinoma	HIF1A;VEGFA;MET;CDC42;EGLN3;CRK;PAK1
37	4672	0.0257423	2.927419355	2.314486	6	39	Intestinal immune network for IgA production	CXCR4;CD40LG;IL15;CCR9;CXCL12;TNFSF13B
38	4062	0.038748278	1.834493956	7.596262	13	128	Chemokine signaling pathway	STAT1;CXCR4;CCL4;IL8;CCL2;CCR9;CXCL12;NFKB1;CDC42;CHUK;CRK;CXCL13;PAK1

KEGG enrichment of Tongcheng specific DEmiRNAs' target genes

	KEGGID	Pvalue	OddsRatio	ExpCount	Count	Size	Term	Symbols
1	5220	0.000275496	15.28727273	0.328037	4	54	Chronic myeloid leukemia	TGFBR1;BCL2L1;CDKN1A;SHC1
2	5219	0.000655062	21.21070234	0.176168	3	29	Bladder cancer	IL8;MMP9;CDKN1A
3	4510	0.001274813	7.320540156	0.844393	5	139	Focal adhesion	ITGB3;COL5A3;BCL2;SHC1;ITGB4
4	5142	0.001692087	9.136911281	0.528505	4	87	Chagas disease (American trypanosomiasis)	TGFBR1;IL8;SERPINE1;IL1B
5	5200	0.002600469	5.153846154	1.457944	6	240	Pathways in cancer	TGFBR1;IL8;BCL2L1;MMP9;BCL2;CDKN1A
6	4610	0.005414899	9.604118993	0.364486	3	60	Complement and coagulation cascades	SERPINE1;PLAT;PLAUR
7	4512	0.00648769	8.965787598	0.388785	3	64	ECM-receptor interaction	ITGB3;COL5A3;ITGB4
8	4210	0.008320854	8.151200519	0.425234	3	70	Apoptosis	IL1B;BCL2L1;BCL2
9	5215	0.008653139	8.029411765	0.431308	3	71	Prostate cancer	BCL2;CDKN1A;CREB5
10	5323	0.010060312	7.576086957	0.455607	3	75	Rheumatoid arthritis	MMP3;IL8;IL1B
11	5146	0.010431806	7.470518166	0.461682	3	76	Amoebiasis	IL8;IL1B;COL5A3
12	4142	0.015995916	6.321536906	0.540654	3	89	Lysosome	M6PR;AP1S2;CTSC
13	4722	0.016481344	6.247376312	0.546729	3	90	Neurotrophin signaling pathway	NTRK3;BCL2;SHC1
14	4380	0.017477138	6.104054714	0.558879	3	92	Osteoclast differentiation	TGFBR1;ITGB3;IL1B
15	5014	0.017670909	10.99479167	0.206542	2	34	Amyotrophic lateral sclerosis (ALS)	BCL2L1;BCL2
16	4621	0.02291196	9.497747748	0.236916	2	39	NOD-like receptor signaling pathway	IL8;IL1B
17	5214	0.027510567	8.56300813	0.261215	2	43	Glioma	CDKN1A;SHC1
18	5144	0.029938774	8.160852713	0.273364	2	45	Malaria	IL8;IL1B
19	5210	0.029938774	8.160852713	0.273364	2	45	Colorectal cancer	TGFBR1;BCL2
20	5100	0.029938774	8.160852713	0.273364	2	45	Bacterial invasion of epithelial cells	SHC1;ARPC1B
21	4520	0.033735907	7.623188406	0.291589	2	48	Adherens junction	TGFBR1;SNAI2
22	5212	0.037712274	7.151360544	0.309813	2	51	Pancreatic cancer	TGFBR1;BCL2L1
23	4115	0.041860674	6.733974359	0.328037	2	54	p53 signaling pathway	SERPINE1;CDKN1A
24	5412	0.044718375	6.481481481	0.340187	2	56	Arrhythmogenic right ventricular cardiomyopathy (ARVC)	ITGB3;ITGB4

Supplementary Table S5. Accession number of the genome of the eight PRRSV strains

Strains	NCBI Accession Number
WUH3	HM853673.2
VR2332	EF536003.1
Lelystad	M96262.2
JXA1	EF112445.1
09HUN1	JF268673.1
09HUB1	JF268682.1
CH-1a	AY032626.1
EuroPRRSV	AY366525.1

Supplementary Table S6. 14 microRNAs predicted to bind to conserved regions of WUH3 genome

	PRRSV genome site		T0 vs T3	T0 vs T5	T0 vs T7	L0 vs L3	L0 vs L5	L0 vs L7
microRNA	Start	End	Log2FC					
ssc-miR-26a	185	207	-0.14823	0.424783	-0.39112	-0.23401	0.009585	-0.62601
ssc-miR-1277	6768	6790	—	—	—	—	—	—
ssc-miR-140-3p	7614	7637	0.615725	-0.5984	1.19406	0.559142	0.9037	-0.10802
ssc-miR-1306-3p	7600	7621	0.611794	1.57762	0.881654	0.809061	0.818662	0.543336
ssc-miR-499-3p	10013	10035	—	—	—	—	—	—
ssc-miR-489	10014	10036	—	—	—	—	—	—
ssc-miR-135	13205	13228	—	—	—	—	—	—
ssc-miR-31	13436	13458	-0.25285	0.097938	-0.48175	-2.06351	-1.41166	-3.04356
ssc-miR-299	14770	14791	—	—	—	-0.38932	-2.47508	1.84716
ssc-miR-103	14855	14878	0.032417	-0.02493	-0.3675	-0.46783	-0.28329	-1.77382
ssc-miR-107	14855	14878	0.023185	-0.06187	-0.42517	-0.48439	-0.35333	-1.84131
ssc-miR-338	14850	14872	0.012317	0.391919	1.5513	-0.19452	1.1819	1.04386
ssc-miR-376b	14828	14850	—	—	—	—	—	—
ssc-miR-2320-5p	14847	14869	-2.20445	-0.18102	-0.61865	-2.13967	-1.05893	-2.3478

"_": no detection

Supplementary Table S7. MicroRNAs editing level in the lungs of Tongcheng and Landrace

microRNA_name	Location_inside_pre_miRNA	Mismatch_type	T0	T3	T5	T7
ssc-let-7a-1	6	GU	0.470588	0.8	0.875	0.631579
ssc-let-7a-2	19	UG	0.017794	0.024753	NULL	NULL
ssc-let-7a-2	6	UG	NULL	NULL	NULL	0.333333
ssc-let-7a-2	7	GC	NULL	NULL	NULL	0.1875
ssc-let-7a-2	9	GA	0.682927	0.677419	0.404255	NULL
ssc-let-7a-2	9	GU	NULL	NULL	NULL	0.255814
ssc-let-7c	14	GU	NULL	NULL	0.333333	0.115385
ssc-let-7c	15	UC	0.110583	0.142077	0.105209	0.207739
ssc-let-7d	10	AC	0.165049	0.268293	0.15	0.217391
ssc-let-7d	16	UG	0.007986	0.009044	NULL	NULL
ssc-let-7d	18	GU	0.002624	0.002154	NULL	NULL
ssc-let-7d	19	UC	NULL	NULL	0.006117	NULL
ssc-let-7d	19	UG	0.007068	0.005983	NULL	NULL
ssc-let-7d	9	GA	NULL	0.8	NULL	NULL
ssc-let-7e	15	UG	0.014308	0.012894	NULL	NULL
ssc-let-7e	8	GC	NULL	NULL	NULL	0.526316
ssc-let-7e	8	GU	NULL	NULL	0.5	NULL
ssc-let-7e	9	CG	NULL	0.121622	0.118421	NULL
ssc-let-7f-1	6	GU	0.571429	0.583333	0.461538	0.266667
ssc-let-7f-2	10	GC	NULL	0.088235	NULL	0.315789
ssc-let-7g	11	GU	0.002044	0.0015	NULL	NULL
ssc-let-7g	12	UA	NULL	NULL	0.004595	NULL
ssc-let-7g	14	GU	0.004408	0.005567	0.00629	NULL
ssc-let-7g	18	GC	NULL	NULL	NULL	0.00270237
ssc-let-7g	19	UG	0.002471	NULL	NULL	NULL
ssc-let-7g	3	CU	NULL	0.004146	0.007706	0.00578313
ssc-let-7g	7	GU	NULL	0.001475	NULL	NULL
ssc-let-7g	9	UC	NULL	NULL	NULL	0.00153175
ssc-let-7g	9	UG	0.009139	0.009835	NULL	NULL
ssc-let-7i	11	UG	0.009738	0.010742	NULL	NULL
ssc-let-7i	13	GU	0.001908	NULL	NULL	NULL
ssc-let-7i	14	UC	NULL	NULL	0.006866	NULL
ssc-let-7i	14	UG	0.00458	0.003202	NULL	NULL
ssc-let-7i	16	GU	0.004703	0.006422	0.007012	0.00177173
ssc-let-7i	17	UG	0.031147	0.023377	0.002884	NULL
ssc-let-7i	4	GU	1	0.8125	0.87013	0.671875
ssc-let-7i	5	CA	0.04902	NULL	NULL	NULL
ssc-let-7i	5	CU	NULL	NULL	NULL	0.0131868
ssc-mir-100	15	AC	NULL	NULL	0.888889	NULL
ssc-mir-10a	15	GU	0.002283	0.002092	NULL	NULL
ssc-mir-10a	16	UG	0.016285	0.012849	NULL	NULL
ssc-mir-10a	17	AC	NULL	NULL	0.006749	NULL
ssc-mir-10a	19	AU	NULL	NULL	0.006099	NULL
ssc-mir-10a	8	AC	NULL	NULL	NULL	0.269231
ssc-mir-10a	9	UG	0.001572	NULL	NULL	NULL
ssc-mir-10b	17	UG	0.037464	0.023673	NULL	NULL
ssc-mir-10b	18	AU	NULL	NULL	0.009904	NULL
ssc-mir-122	14	GA	NULL	NULL	NULL	0.363636
ssc-mir-122	14	GC	0.714286	0.5	NULL	NULL
ssc-mir-122	19	GU	0.006385	0.003526	NULL	NULL
ssc-mir-125a	18	GU	0.032819	0.023894	NULL	NULL
ssc-mir-125a	19	AU	NULL	NULL	0.007655	NULL
ssc-mir-125b-1	16	GU	0.018005	0.012504	NULL	NULL
ssc-mir-125b-1	17	AC	NULL	NULL	0.008311	NULL
ssc-mir-125b-1	19	CG	NULL	NULL	0.005965	NULL
ssc-mir-126	19	UG	NULL	NULL	0.006107	NULL
ssc-mir-126	6	GC	NULL	1	NULL	0.75
ssc-mir-1307	17	GU	0.025981	0.036375	NULL	NULL
ssc-mir-1307	18	AC	NULL	NULL	0.009351	NULL
ssc-mir-139	14	UG	0.062176	0.056838	NULL	NULL
ssc-mir-139	15	GC	NULL	NULL	0.008475	NULL
ssc-mir-142	19	UG	0.019368	0.019839	NULL	NULL
ssc-mir-145	15	GC	0.517241	0.5	0.461538	0.7
ssc-mir-145	16	GU	NULL	0.001852	NULL	NULL
ssc-mir-145	17	UG	0.008852	0.009205	NULL	NULL
ssc-mir-148b	13	GU	0.034783	NULL	NULL	NULL
ssc-mir-150-2	19	CU	NULL	NULL	0.006326	NULL
ssc-mir-15a	11	GA	0.76	NULL	NULL	NULL
ssc-mir-15b	19	GA	0.714286	NULL	0.583333	NULL
ssc-mir-16-2	7	CG	NULL	NULL	NULL	0.136364
ssc-mir-16-2	8	UA	NULL	NULL	0.428571	NULL

ssc-mir-16-2	8	UG	NULL	NULL	NULL	0.695652
ssc-mir-17	14	GU	0.005051	0.006539	NULL	NULL
ssc-mir-17	15	UG	0.008418	0.006036	NULL	NULL
ssc-mir-17	18	UC	NULL	NULL	0.005535	NULL
ssc-mir-17	9	UA	0.095588	NULL	NULL	NULL
ssc-mir-181a-2	7	AC	NULL	NULL	NULL	0.222222
ssc-mir-181a-2	9	GU	NULL	NULL	NULL	0.434783
ssc-mir-181c	14	GA	NULL	0.444444	0.333333	NULL
ssc-mir-181d	17	UC	NULL	NULL	0.004837	NULL
ssc-mir-181d	19	GU	0.033623	0.029934	0.005662	NULL
ssc-mir-1839	14	GU	NULL	0.012048	NULL	NULL
ssc-mir-1839	15	UG	0.077273	0.062432	NULL	NULL
ssc-mir-183	17	UG	NULL	NULL	0.008029	NULL
ssc-mir-185	10	UA	0.318182	NULL	NULL	NULL
ssc-mir-185	10	UC	NULL	0.454545	0.5625	0.705882
ssc-mir-185	19	AU	NULL	NULL	0.006698	NULL
ssc-mir-186	14	CA	0.056911	NULL	NULL	NULL
ssc-mir-186	14	CU	NULL	NULL	NULL	0.0649351
ssc-mir-186	19	GU	0.008041	0.010881	NULL	NULL
ssc-mir-191	13	GU	0.006053	0.00404	NULL	NULL
ssc-mir-191	14	GU	0.001997	NULL	NULL	NULL
ssc-mir-191	17	UC	NULL	NULL	0.005304	NULL
ssc-mir-191	17	UG	0.001848	NULL	NULL	NULL
ssc-mir-191	19	CU	0.002369	NULL	0.006914	NULL
ssc-mir-191	8	GA	0.586207	NULL	NULL	0.269231
ssc-mir-191	8	GC	NULL	0.533333	0.695652	NULL
ssc-mir-192	19	UC	NULL	NULL	0.004259	NULL
ssc-mir-193a	19	GU	NULL	0.020962	NULL	NULL
ssc-mir-195	14	UG	NULL	NULL	NULL	0.428571
ssc-mir-205	17	CG	NULL	NULL	0.01087	NULL
ssc-mir-20a	12	GU	0.009239	NULL	NULL	NULL
ssc-mir-20a	13	UG	0.01413	NULL	NULL	NULL
ssc-mir-20a	16	UC	NULL	NULL	0.004054	NULL
ssc-mir-20a	18	AG	NULL	NULL	0.006283	NULL
ssc-mir-20a	7	CA	0.805556	NULL	NULL	NULL
ssc-mir-21	15	GU	NULL	NULL	NULL	0.210526
ssc-mir-21	17	GA	0.707015	0.652991	0.492826	0.933549
ssc-mir-221	12	UC	NULL	NULL	0.006636	NULL
ssc-mir-221	14	CG	NULL	NULL	0.004304	NULL
ssc-mir-221	18	GU	0.027635	0.024422	NULL	NULL
ssc-mir-221	7	UG	0.012189	0.009004	NULL	NULL
ssc-mir-221	8	GU	0.006089	0.005658	NULL	NULL
ssc-mir-2320	18	GU	0.014925	NULL	NULL	NULL
ssc-mir-2320	19	GU	NULL	NULL	0.007303	NULL
ssc-mir-23b	16	UG	0.041943	0.03834	NULL	NULL
ssc-mir-24-1	13	UG	0.048781	0.05283	NULL	NULL
ssc-mir-26a	12	GA	0.590909	0.5	0.230769	NULL
ssc-mir-26a	12	GC	NULL	NULL	NULL	0.0952381
ssc-mir-26a	13	AC	0.537549	0.62069	0.877941	0.789286
ssc-mir-26a	14	UG	0.001496	NULL	NULL	NULL
ssc-mir-26a	15	UG	NULL	0.002921	NULL	NULL
ssc-mir-26a	19	GU	0.00225	NULL	NULL	NULL
ssc-mir-30e	10	GU	0.004072	0.003093	NULL	NULL
ssc-mir-30e	17	UC	NULL	NULL	0.004897	NULL
ssc-mir-30e	18	CU	NULL	NULL	0.002253	NULL
ssc-mir-30e	19	CU	0.010381	0.004501	0.01048	0.00467219
ssc-mir-30e	8	CA	0.642857	NULL	NULL	NULL
ssc-mir-31	10	GA	NULL	NULL	0.266667	NULL
ssc-mir-31	10	GU	NULL	NULL	NULL	0.8
ssc-mir-31	12	GU	0.005027	NULL	NULL	NULL
ssc-mir-31	19	UC	NULL	NULL	0.005915	NULL
ssc-mir-335	19	GU	0.010764	0.00765	NULL	NULL
ssc-mir-34c-2	10	UC	0.199546	0.195307	0.197084	0.229077
ssc-mir-34c-2	15	AC	NULL	NULL	NULL	0.00246844
ssc-mir-34c-2	17	UG	0.006742	0.008202	NULL	NULL
ssc-mir-34c-2	18	GU	0.006149	0.004729	NULL	NULL
ssc-mir-34c-2	19	UC	NULL	NULL	0.006298	NULL
ssc-mir-34c-2	19	UG	0.00848	0.005262	NULL	NULL
ssc-mir-361	16	CG	NULL	NULL	0.005505	NULL
ssc-mir-361	5	CA	0.444444	NULL	NULL	NULL
ssc-mir-362	18	GU	0.018315	NULL	NULL	NULL
ssc-mir-363-2	15	UG	0.09375	0.094017	NULL	NULL
ssc-mir-423	13	GU	0.006239	0.009301	NULL	NULL
ssc-mir-423	15	GU	0.002231	NULL	NULL	0.00198923
ssc-mir-423	18	AC	NULL	NULL	0.008836	NULL
ssc-mir-423	8	GC	NULL	NULL	NULL	0.823529

ssc-mir-423	9	CA	NULL	0.181818	NULL	NULL
ssc-mir-425	11	GU	NULL	NULL	NULL	0.428571
ssc-mir-425	15	GU	0.023823	0.024848	0.003777	NULL
ssc-mir-451	11	AC	0.5	NULL	NULL	0.142857
ssc-mir-451	12	GU	0.428571	0.333333	NULL	0.566667
ssc-mir-451	13	CA	0.411765	0.509804	0.438202	0.139535
ssc-mir-451	19	GU	0.004116	0.002506	NULL	NULL
ssc-mir-497	18	CG	NULL	NULL	0.005788	NULL
ssc-mir-497	19	UG	0.005829	NULL	NULL	NULL
ssc-mir-497	7	CA	0.6	NULL	NULL	NULL
ssc-mir-497	9	AG	0.014583	0.011633	0.013121	0.0170584
ssc-mir-505	16	GU	0.03681	NULL	NULL	NULL
ssc-mir-532	19	GU	0.009319	0.008627	NULL	NULL
ssc-mir-664	18	AG	NULL	NULL	NULL	0.0108814
ssc-mir-664	19	GU	0.007177	NULL	NULL	NULL
ssc-mir-744	15	UC	NULL	NULL	0.008383	NULL
ssc-mir-744	18	GU	NULL	NULL	NULL	0.00530705
ssc-mir-744	19	GU	NULL	0.026385	NULL	NULL
ssc-mir-92a-1	18	GU	0.055172	NULL	NULL	NULL
ssc-mir-92b	13	GU	NULL	0.008344	NULL	NULL
ssc-mir-92b	17	GU	0.007534	0.00706	NULL	NULL
ssc-mir-92b	18	GA	NULL	NULL	0.008157	NULL
ssc-mir-98	12	UC	NULL	NULL	0.005362	NULL
ssc-mir-98	12	UG	0.004202	NULL	NULL	NULL
ssc-mir-98	14	AU	NULL	0.004186	0.0044	NULL
ssc-mir-98	18	GU	0.004202	NULL	NULL	NULL
ssc-mir-98	3	GC	0.5	0.5	0.357143	NULL
ssc-mir-98	9	UG	0.011555	0.009604	NULL	NULL
ssc-mir-99a	11	AC	0.026432	0.022523	0.016393	0.0584795
ssc-mir-99a	12	AG	0.064173	0.055661	0.0659	0.0638892
ssc-mir-99a	17	GU	0.004581	NULL	NULL	NULL
ssc-mir-99a	18	UG	0.004373	0.005769	NULL	NULL
ssc-mir-99b	12	GU	0.00328	0.002159	NULL	NULL
ssc-mir-99b	13	UG	0.005193	0.006323	NULL	NULL
ssc-mir-99b	15	GU	NULL	0.002673	0.006446	NULL
ssc-mir-99b	17	AU	NULL	NULL	0.006634	NULL
ssc-mir-99b	6	CA	NULL	NULL	NULL	0.25

**microRNA_name**	**Location_inside_pre_miRNA**	**Mismatch_type**	**L0**	**L3**	**L5**	**L7**
ssc-let-7a-1	6	GU	0.580645	0.625	0.368421	0.667
ssc-let-7a-1	7	UG	NULL	NULL	NULL	0.018
ssc-let-7a-2	19	UG	0.010091	NULL	NULL	0.027
ssc-let-7a-2	6	UA	NULL	NULL	NULL	0.375
ssc-let-7a-2	7	GC	NULL	NULL	0.136364	NULL
ssc-let-7a-2	7	GU	NULL	NULL	NULL	0.077
ssc-let-7a-2	8	GA	0.25	NULL	NULL	NULL
ssc-let-7a-2	9	GA	0.77381	0.211538	0.216216	0.258
ssc-let-7c	14	GU	0.24	0.181818	0.096774	NULL
ssc-let-7c	15	UC	0.092025	0.13807	0.157025	0.133
ssc-let-7d	10	AC	0.141509	0.216049	0.267101	0.188
ssc-let-7d	16	UG	0.005959	NULL	NULL	NULL
ssc-let-7d	18	GU	0.001647	NULL	NULL	NULL
ssc-let-7d	19	UG	0.00754	NULL	NULL	NULL
ssc-let-7d	9	GA	0.727273	NULL	NULL	NULL
ssc-let-7e	13	GU	NULL	0.001487	NULL	NULL
ssc-let-7e	15	UG	0.009318	NULL	NULL	NULL
ssc-let-7e	8	GC	NULL	0.25	0.157895	0.2
ssc-let-7e	9	CG	0.153226	0.065934	0.086539	NULL
ssc-let-7f-1	6	GU	0.272727	0.368421	0.333333	0.286
ssc-let-7f-1	7	AC	NULL	0.114286	0.049505	NULL
ssc-let-7f-2	10	GC	0.075	0.095238	0.061728	0.143
ssc-let-7g	11	GU	0.001495	NULL	NULL	NULL
ssc-let-7g	14	GU	0.004522	NULL	NULL	NULL
ssc-let-7g	19	UG	0.001576	NULL	NULL	NULL
ssc-let-7g	3	CA	0.007998	NULL	0.011436	NULL
ssc-let-7g	3	CU	NULL	0.00578	0.006321	NULL
ssc-let-7g	7	GU	NULL	0.001463	0.001231	NULL
ssc-let-7g	9	UC	NULL	0.001409	0.001237	NULL
ssc-let-7g	9	UG	0.006082	NULL	NULL	NULL
ssc-let-7i	11	UG	0.00611	NULL	NULL	NULL
ssc-let-7i	14	UG	0.003856	NULL	NULL	NULL
ssc-let-7i	16	GU	0.005017	0.001997	NULL	0.002
ssc-let-7i	17	UG	0.036183	NULL	0.003088	NULL
ssc-let-7i	4	GU	0.971014	0.676471	0.823009	0.579
ssc-let-7i	5	CA	0.073034	NULL	NULL	NULL
ssc-mir-100	15	AC	0.6	0.666667	0.454545	NULL
ssc-mir-106a	12	UA	NULL	NULL	NULL	0.5

ssc-mir-10a	15	GU	0.001669	NULL	NULL	NULL
ssc-mir-10a	16	UG	0.018618	NULL	NULL	NULL
ssc-mir-10a	8	AC	0.023697	0.048387	0.020036	0.077
ssc-mir-10b	17	UG	0.031159	NULL	NULL	NULL
ssc-mir-122	14	GC	0.466667	NULL	NULL	NULL
ssc-mir-122	19	GU	0.008254	NULL	NULL	NULL
ssc-mir-125a	18	GU	0.02538	NULL	NULL	NULL
ssc-mir-125b-1	16	GU	0.019582	NULL	NULL	NULL
ssc-mir-125b-1	8	GC	0.583333	NULL	NULL	NULL
ssc-mir-126	6	GC	NULL	0.625	0.666667	1
ssc-mir-126	7	GA	0.722222	NULL	NULL	NULL
ssc-mir-1307	17	GU	0.032496	NULL	NULL	NULL
ssc-mir-139	14	UG	0.089207	NULL	NULL	NULL
ssc-mir-142	19	UG	0.0221	NULL	NULL	NULL
ssc-mir-145	14	CG	NULL	0.6	0.6	NULL
ssc-mir-145	15	GA	0.736842	NULL	NULL	NULL
ssc-mir-145	15	GC	NULL	0.310345	0.326923	0.333
ssc-mir-145	16	GU	0.003118	NULL	NULL	NULL
ssc-mir-145	17	UG	0.012464	NULL	NULL	NULL
ssc-mir-1468	15	GU	0.009292	NULL	NULL	NULL
ssc-mir-148b	13	GU	0.034826	NULL	NULL	NULL
ssc-mir-148b	14	UG	0.039801	NULL	NULL	NULL
ssc-mir-15a	11	GA	0.82	NULL	NULL	NULL
ssc-mir-15b	16	AG	1	NULL	NULL	NULL
ssc-mir-15b	19	GA	0.470588	NULL	NULL	NULL
ssc-mir-15b	19	GC	NULL	NULL	0.375	0.5
ssc-mir-16-1	5	CA	1	NULL	NULL	NULL
ssc-mir-16-2	7	CG	0.307692	NULL	NULL	NULL
ssc-mir-16-2	8	UG	0.357143	NULL	0.7	NULL
ssc-mir-17	14	GU	0.004466	NULL	NULL	NULL
ssc-mir-17	15	UG	0.004631	NULL	NULL	NULL
ssc-mir-17	9	UA	0.084375	NULL	NULL	NULL
ssc-mir-181a-2	7	AU	NULL	0.571429	0.666667	0.4
ssc-mir-181a-2	9	GU	0.714286	NULL	0.375	NULL
ssc-mir-181c	14	GA	0.5	NULL	NULL	NULL
ssc-mir-181c	14	GU	NULL	NULL	NULL	0.15
ssc-mir-181d	19	GU	0.037789	NULL	NULL	NULL
ssc-mir-1839	15	UG	0.091392	NULL	NULL	NULL
ssc-mir-185	10	UC	0.714286	0.785714	0.583333	0.9
ssc-mir-186	14	CA	0.033333	NULL	NULL	NULL
ssc-mir-186	19	GU	0.007944	NULL	NULL	NULL
ssc-mir-18a	19	UG	0.045455	NULL	NULL	NULL
ssc-mir-191	13	GU	0.005911	NULL	NULL	NULL
ssc-mir-191	14	GU	0.00177	NULL	NULL	NULL
ssc-mir-191	17	UG	0.001809	NULL	NULL	NULL
ssc-mir-191	19	CU	0.001704	0.001765	NULL	0.002
ssc-mir-191	7	GC	NULL	NULL	0.428571	NULL
ssc-mir-191	8	GA	0.75	NULL	NULL	NULL
ssc-mir-191	8	GC	NULL	0.571429	0.535714	0.471
ssc-mir-192	12	UG	0.006303	NULL	NULL	NULL
ssc-mir-195	15	CA	0.666667	NULL	NULL	NULL
ssc-mir-195	15	CG	NULL	NULL	NULL	0.188
ssc-mir-20a	12	GU	0.006182	NULL	NULL	NULL
ssc-mir-20a	13	UG	0.008655	NULL	NULL	NULL
ssc-mir-20a	7	CA	0.867925	NULL	NULL	NULL
ssc-mir-20a	7	CG	NULL	NULL	NULL	0.333
ssc-mir-21	15	GA	NULL	0.2	0.24	0.318
ssc-mir-21	17	GA	0.645028	0.569304	0.570619	0.568
ssc-mir-221	18	GU	0.031613	NULL	NULL	NULL
ssc-mir-221	7	UG	0.014194	NULL	NULL	NULL
ssc-mir-2320	18	GU	0.014184	NULL	NULL	NULL
ssc-mir-23b	16	UG	0.056078	NULL	NULL	NULL
ssc-mir-24-1	13	UG	0.03629	NULL	NULL	NULL
ssc-mir-26a	10	UG	NULL	NULL	0.133333	0.188
ssc-mir-26a	12	GA	0.858586	NULL	0.16129	NULL
ssc-mir-26a	13	AC	0.389864	0.878788	0.892157	0.889
ssc-mir-26a	15	UG	0.002443	NULL	NULL	NULL
ssc-mir-26a	19	GU	0.001708	NULL	NULL	NULL
ssc-mir-27a	14	AG	NULL	0.014025	0.01737	0.02
ssc-mir-27b	13	GU	0.008035	NULL	NULL	NULL
ssc-mir-30e	10	GU	0.00498	NULL	NULL	NULL
ssc-mir-30e	19	CU	0.009805	0.004431	0.004127	0.004
ssc-mir-30e	8	CA	0.777778	NULL	NULL	NULL
ssc-mir-31	10	GA	0.857143	NULL	NULL	NULL
ssc-mir-31	12	GU	0.004469	NULL	NULL	NULL
ssc-mir-31	19	UG	0.004105	NULL	NULL	NULL

ssc-mir-335	14	GA	0.5	NULL	NULL	NULL
ssc-mir-335	19	GU	0.008536	NULL	NULL	NULL
ssc-mir-34c-2	10	UC	0.338308	0.315442	0.313758	0.315
ssc-mir-34c-2	12	GU	0.001269	NULL	NULL	NULL
ssc-mir-34c-2	15	AC	NULL	0.002313	0.001836	NULL
ssc-mir-34c-2	17	UG	0.00559	NULL	NULL	NULL
ssc-mir-34c-2	18	GU	0.005155	NULL	NULL	NULL
ssc-mir-34c-2	19	UG	0.007428	NULL	NULL	NULL
ssc-mir-361	5	CA	0.571429	NULL	0.25	NULL
ssc-mir-362	18	GU	0.01019	NULL	NULL	NULL
ssc-mir-363-2	14	GU	0.038835	NULL	NULL	NULL
ssc-mir-363-2	15	UG	0.057692	NULL	NULL	NULL
ssc-mir-374a	9	AC	0.125	NULL	NULL	NULL
ssc-mir-374b	8	GA	0.5	NULL	NULL	NULL
ssc-mir-423	13	GU	0.007381	NULL	NULL	NULL
ssc-mir-423	15	GU	0.002092	NULL	NULL	NULL
ssc-mir-423	8	GU	0.5	NULL	NULL	NULL
ssc-mir-423	9	CA	0.084746	0.166667	NULL	NULL
ssc-mir-425	11	GA	0.75	0.5	NULL	0.6
ssc-mir-425	15	GU	0.028584	0.005127	0.00488	NULL
ssc-mir-451	12	GU	0.545455	0.407407	0.368421	0.375
ssc-mir-451	13	CA	0.546512	0.336842	0.345794	0.295
ssc-mir-451	19	GU	0.00371	NULL	NULL	0.002
ssc-mir-497	19	UG	0.011906	NULL	NULL	NULL
ssc-mir-497	7	CA	0.857143	NULL	NULL	NULL
ssc-mir-497	9	AG	0.016511	0.010574	0.012334	0.009
ssc-mir-532	19	GU	0.007603	0.001933	0.001796	NULL
ssc-mir-664	18	AG	0.008215	0.012223	0.010022	0.014
ssc-mir-744	19	GU	0.011587	NULL	NULL	NULL
ssc-mir-744	6	GC	0.6	NULL	NULL	NULL
ssc-mir-92b	17	GU	0.00707	NULL	NULL	NULL
ssc-mir-98	12	UG	0.004765	NULL	NULL	NULL
ssc-mir-98	15	GU	0.004032	NULL	NULL	NULL
ssc-mir-98	18	GU	0.005865	NULL	NULL	NULL
ssc-mir-98	3	GA	0.583333	NULL	NULL	NULL
ssc-mir-98	3	GC	NULL	NULL	0.75	0.727
ssc-mir-98	9	UG	0.008248	NULL	NULL	NULL
ssc-mir-99a	11	AC	NULL	0.019157	0.019002	0.022
ssc-mir-99a	12	AG	0.056224	0.055157	0.052545	0.057
ssc-mir-99a	17	GU	0.002153	NULL	NULL	0.002
ssc-mir-99a	18	UG	0.003362	NULL	NULL	NULL
ssc-mir-99b	12	GU	0.002271	0.002098	NULL	0.004
ssc-mir-99b	13	UG	0.002994	NULL	NULL	NULL
ssc-mir-99b	15	GU	0.003406	NULL	NULL	NULL
ssc-mir-99b	5	CU	1	NULL	NULL	NULL
ssc-mir-99b	6	CA	0.214286	NULL	0.263158	NULL

“NULL”: no detection

Supplementary Table S8 | MicroRNA editing sites number

	L0	L3	L5	L7	Total	T0	T3	T5	T7	Total
microRNA	65	31	36	26	67	52	46	46	32	65
MicroRNA editing sites	133	47	48	40	150	101	85	72	48	168

Supplementary Table S9 | Primers for qRT-PCR

miRNAs	Primers
miRNA-2320-5p	TGGCACAGGGTCCAGCTGTC
miRNA-374a-3p	ccgggCTTATCAGGTTGTATTGTAATT
Novel miRNA (5:5388562-5388582)	aaATGCGGAACCTGCGGATAC
miRNA-204	aacTTCCCTTTGTCATCCTATGCCT
miRNA-148a-3p	agcggTCAGTGCACTACAGAACTTTGT

miRNA-424-5p	agCAGCAGCAATTCATGTTTTGAA
U6-F	CTCGCTTCGGCAGCACA
U6-R	AACGCTTCACGAATTTGCGT
RT-primer	GCGAGCACAGAATTAATACGACTCACTATAGGTTTTTTTTTTTT
Uni-miR-R primer	GCGAGCACAGAATTAATACGACTCAC

Supplementary Table S10 | The overlapped DEmiRNAs between the two breeds

	0 dpi vs. 3 dpi		0 dpi vs. 5 dpi		0 dpi vs. 7 dpi	
	Tongcheng	Landrace	Tongcheng	Landrace	Tongcheng	Landrace
Number of DEmiRNAs	52	72	62	75	76	109
Overlapped DEmiRNAs	34		35		52	
The ratio of overlapped DEmiRNAs	34/52 (65.38%)	34/72 (47.22%)	35/62 (56.45%)	35/75 (46.67%)	52/76 (68.42%)	52/109 (47.71%)

The details for the identification of microRNA editing sites:

1. Filtering reads with low quality and remove adaptors

   1). Using the fastx_clipper in FASTX-Toolkit to remove adaptors.

   2). Using FASTX-Toolkit to trim the bases have a quality less than 20 and abandon reads that have more than 50% bases with a less than 20 quality.

2. Align clean reads on reference genome using bowtie

   We used Bowtie to map reads on reference genome and extract the unique map location reads to perform further analysis.

3. Mapping the mis-matches against the Pre-miRNA sequences

   1). We downloaded the Sus_hairpin sequences from miRBase 19 databases to mapping them on pig reference genome. And, we can get the locations of the pre-miRNAs.

2). Next, we used Analyze_mutation.pl script with the pre-miRNA mapping output and Bowtie output files as input files to get the mutations in pre-miRNA sequences.

4. Remove sequencing errors using binomial statistics

We used Binomial_analysis.pl with the output file from the previous step as input to perform binomial statistics. The P-value was corrected using Bonferroni.

5. Remove the known SNPs

As the mis-matches we detected might be the known SNPs, to avoid this kind of impact, we downloaded the known SNPs of pigs from Ensembl. We used customized perl script to remove these known SNPs from our results.
